# Protocol for a pilot randomised controlled trial to evaluate integrated support from pharmacist independent prescriber and third sector worker for people experiencing homelessness: the PHOENIx community pharmacy study

**DOI:** 10.1186/s40814-023-01261-x

**Published:** 2023-02-23

**Authors:** Vibhu Paudyal, Richard Lowrie, Frances S. Mair, Lee Middleton, Versha Cheed, Jennifer Hislop, Andrea Williamson, Nigel Barnes, Catherine Jolly, Karen Saunders, Natalie Allen, Parbir Jagpal, George Provan, Steven Ross, Carole Hunter, Sarah Tearne, Andrew McPherson, Helena Heath, Cian Lombard, Adnan Araf, Emily Dixon, Amy Hatch, Jane Moir, Shabana Akhtar

**Affiliations:** 1 School of Pharmacy, College of Medical and Dental Sciences, University of Birmingham, Birmingham, UK; 2 Pharmacy Services, NHS Greater Glasgow and Clyde, Scotland, UK; 3 Homeless Health / Research and Development, NHS Greater Glasgow and Clyde, Glasgow, G76 7AT Scotland UK; 4 General Practice and Primary Care, Institute of Health and Wellbeing, College of Medical, Veterinary and Life Sciences, University of Glasgow, Glasgow, UK; 5 Birmingham Clinical Trials Unit, University of Birmingham, Birmingham, UK; 6 Healthcare Improvement Scotland, Edinburgh, UK; 7 Undergraduate Medical School, School of Medicine, Dentistry and Nursing, College of Medical, Veterinary and Life Sciences, University of Glasgow, Glasgow, UK; 8 NHS Birmingham and Solihull Mental Health Foundations Trust, Birmingham, UK; 9 Institute of Applied Health Research, University of Birmingham, Birmingham, UK; 10 Office for Health Improvement and Disparities, Department of Health and Social Care, Birmingham, UK; 11 SIFA Fireside, Birmingham, UK; 12 Simon Community Scotland, Glasgow, UK; 13 Glasgow City HSCP, Alcohol and Drug Recovery Services, Glasgow, UK

**Keywords:** Homelessness, Health inequality, Integrated care, Pharmacist independent prescriber

## Abstract

**Background:**

People experiencing homelessness (PEH) have complex health and social care needs and most die in their early 40 s. PEH frequently use community pharmacies; however, evaluation of the delivery of structured, integrated, holistic health and social care intervention has not been previously undertaken in community pharmacies for PEH. PHOENIx (Pharmacy Homeless Outreach Engagement Non-medical Independent prescribing Rx) has been delivered and tested in Glasgow, Scotland, by NHS pharmacist independent prescribers and third sector homelessness support workers offering health and social care intervention in low threshold homeless drop-in venues, emergency accommodation and emergency departments, to PEH. Building on this work, this study aims to test recruitment, retention, intervention adherence and fidelity of community pharmacy-based PHOENIx intervention.

**Methods:**

Randomised, multi-centre, open, parallel-group external pilot trial. A total of 100 PEH aged 18 years and over will be recruited from community pharmacies in Glasgow and Birmingham. PHOENIx intervention includes structured assessment in the community pharmacy of health, housing, benefits and activities, in addition to usual care, through weekly visits lasting up to six months. A primary outcome is whether to proceed to a definitive trial based on pre-specified progression criteria. Secondary outcomes include drug/alcohol treatment uptake and treatment retention; overdose rates; mortality and time to death; prison/criminal justice encounters; healthcare utilisation; housing tenure; patient-reported measures and intervention acceptability. Analysis will include descriptive statistics of recruitment and retention rates. Process evaluation will be conducted using Normalisation Process Theory. Health, social care and personal resource use data will be identified, measured and valued.

**Discussion:**

If the findings of this pilot study suggest progression to a definitive trial, and if the definitive trial offers positive outcomes, it is intended that PHOENIx will be a publicly funded free-to-access service in community pharmacy for PEH. The study results will be shared with wider stakeholders and patients in addition to dissemination through medical journals and scientific conferences.

**Trial registration:**

International Clinical Trial Registration ISRCTN88146807.

Approved protocol version 2.0 dated July 19, 2022.

**Supplementary Information:**

The online version contains supplementary material available at 10.1186/s40814-023-01261-x.

## Background

People experiencing homelessness (PEH, including rooflessness, houselessness, insecure or inadequate housing/exempt accommodation tenants) are amongst the most marginalised, destitute and vulnerable groups in the UK. Homelessness is rising in Britain with an estimated 250,000 [1] and 29,000 [2] PEH in England and Scotland, respectively. In addition, urban areas have seen a sharp rise in rough sleeping over recent years [3]. Housing and homelessness have been a high priority for Government at the national and local levels during the COVID-19 pandemic, and continue to be an important societal and public health challenge [3].

Despite most PEH being in their early 40 s, they have on average nine different health problems [4–6], and up to 12 times higher mortality rates [7]. This is on par with people aged 85 years living in their own homes [4]. The average life expectancy for a PEH is 45 years old and one third of deaths are estimated to be preventable if care is received on time [8]. Co-morbid health concerns include physical as well as mental and substance misuse issues. In addition, unstable housing, low income or debt and frequent encounters with the criminal justice system make them socially marginalised.

Most health services operate by appointment, and PEH may live far from their GP, addictions team and/or mental health team, leading to PEH experiencing fragmentation of care. Previous qualitative studies show evidence of stigma and discrimination faced by PEH in the healthcare setting [9]. In addition, contrary to the laws around equity in access to healthcare, PEH are often denied registration at general practice [9] and hence are under-represented in mainstream primary care [10]. Their health conditions hence often remain underdiagnosed, and untreated and they regularly drop out of care. Substance misuse and unstable mental health, housing issues and prioritising immediate safety can also contribute to fluctuating motivation to address health concerns. PEH therefore have a high use of emergency services [11].

PHOENIx [Pharmacy Homeless Outreach Engagement Non-medical Independent prescriber Rx] is a model of care that originated in Glasgow, Scotland that includes a pharmacist independent prescriber working alongside a third sector support worker offering proactive support and outreach services to PEH. A previous feasibility study conducted in Glasgow engaged 124 PEH amongst which 53 (43%) had a new medicine prescribed linked to previously undiagnosed conditions, 8% had a medicine discontinued a further 28% having adjustments in prescribed medicines [12]. In addition, one third of PEH were referred onto other services. In another study the PHOENIx team engaged with 52 patients in streets and low-threshold venues, e.g. homeless shelters, and soup kitchens [13]. Medications were prescribed by PHOENIx pharmacists in 62% of all PEH participants, of which over 60% were new medications. The study also showed improvement in patient engagement with services 85% of those referred attended their follow-up with the PHOENIx team or the referred services [13]. Qualitative evaluation of the PHOENIx service demonstrated positive engagement with the PHOENIx team and valued the services offered in an integrated way [14, 15].

Methods and baseline findings from a previous pilot RCT of PHOENIx with the parallel process and economic evaluation focused on PEH with a history of drug overdose are previously described [6, (Lowrie R, McPherson A, Mair F, et al.: Characteristics of people experiencing homelessness with a recent drug overdose in the PHOENIx pilot randomised controlled trial, unpublished)]; however, a community pharmacy-based study and PHOENIx intervention have not been described.

Community pharmacies in the United Kingdom (UK) provide a range of services that are often accessed by PEH. Over 90% of the people in the UK live within a 20-min walk of a community pharmacy [16]. People can visit a community pharmacy without an appointment. Services offered by community pharmacies include prescription dispensing, needle exchange for those misusing substances, and opioid substitution therapy. However, the interaction is often limited to product supply and the opportunities to offer wider health and social care interventions can be missed [17, 18]. Community pharmacists have shown readiness to take on further roles in supporting the health needs of PEH with 90% of 321 respondents in a recent survey demonstrating readiness to have further involvement in supporting PEH [19]. Making every contact count is embedded in NHS policies [20].

Third sector homeless charity/support staff often have strong working relationships with PEH, providing housing, food, clothing support, and advocacy as well as offering expert advice on benefits and monetary matters. However, third sector staff operate separately and cannot readily share records with the health service without informed consent from patients [21]. Opportunities to capitalise on ‘windows of opportunity’ for PEH who have a fluctuating motivation to engage can be missed. In the health service, assertive outreach to assess and comprehensively address multiple complex health and social care needs is patchy. This study will aim to address these barriers by integrating third sector support staff teams with healthcare professionals.

The aim of this study is to undertake a pilot randomised controlled trial to test recruitment, retention, intervention adherence and fidelity of the PHOENIx intervention delivered in the Community Pharmacy setting. Specific objectives include the evaluation of acceptability of randomisation and adherence with information governance and data collection procedures, and to explore participant and healthcare professional perceptions of the intervention and acceptability of trial procedures. The study will also evaluate whether relevant resource use and health state utility data (as a proxy for quality of life) can be identified, measured and valued appropriately for the purposes of conducting a full economic evaluation to determine the cost-effectiveness of the PHOENIx intervention in a definitive trial.

## Methods

### Patient and public involvement

Patient and public involvement (PPI) informed the development of this proposed pilot study. PPI work has involved patients [17], pharmacists and wider stakeholders in various public engagement events [18]. These included representatives and volunteers from homelessness services, drug and alcohol services, public health and NHS commissioning bodies. In addition, PEH representatives helped the research team to design the protocol and they reviewed patient facing materials for the study. PEH, staff delivering PHOENIx in low threshold homeless venues and emergency homeless accommodation, and stakeholders report the service is effective at case finding and engaging with patients who were reluctant to utilise or physically unable to access existing (mainstream or specialist ‘homeless’) healthcare provision. It helped patients overcome many of the barriers that homeless people commonly face when attempting to access healthcare, enabled immediate diagnosis and prescription of medication, and catalysed and capitalised on windows of opportunity when patients were motivated to address healthcare needs. A number of improvements in health outcomes, including but not limited to medication adherence, were also reported [14]. Participants reported acceptability of being approached for inclusion in a subsequent randomised controlled trial [15].

### Study design and setting

The trial is designed as a randomised, multicentre, open, parallel group external pilot trial alongside economic and qualitative process evaluation.

The trial will be set across two large cities in the UK, Birmingham and Glasgow. The research team comprises diverse expertise in pharmacy practice, health services research, public health, Alcohol and Drug Recovery Services (ADRS), NHS commissioning, trial design, statistics, and third sector including PPI representations. The participating pharmacies are located in town centres and immediate surrounding areas, where PEH are known to be frequent. Initially, two pharmacies in Birmingham and two in Glasgow are recruited with other pharmacies recruited as backup.

### Sample size and statistical power

The sample size calculation is based around two of the main feasibility objectives: rate of recruitment and retention. The study will aim to recruit and randomise 100 participants in total. If the total number of eligible persons is 200, this will allow measurement of the recruitment rate with a 95% confidence interval width of approximately 14%. If 70% of those recruited are followed up in terms of measuring ED visits, this will allow measurement of the rate with a 95% confidence interval width of approximately 18%.

At the point of submission of this study protocol, a total of 61 participants have been recruited into the study.

### Participants (inclusion and exclusion)

Adults ≥ 18 years who are experiencing homelessness will be recruited. Homelessness will cover rooflessness, houselessness, insecure or inadequate housing as per the European Typology of Homelessness and housing exclusion (ETHOS) typology [22]. However, as in our previous work, eligibility does not include those threatened with homelessness [6]. These criteria capture persons staying in homeless shelters, rough sleepers, staying in temporary accommodations such as bed and breakfasts (B&Bs), hostels, squats, or those sofa surfing between family and friends’ houses. Those excluded will include participants living in accommodation with 24-h support which includes in house medical care; intoxicated or (in the opinion of the researcher) posing a safety risk to staff and lacking the capacity to consent as per the Health Research Authority guidance on continued capacity [23].

### Participant identification, informed consent and recruitment

**Fig. 1:**
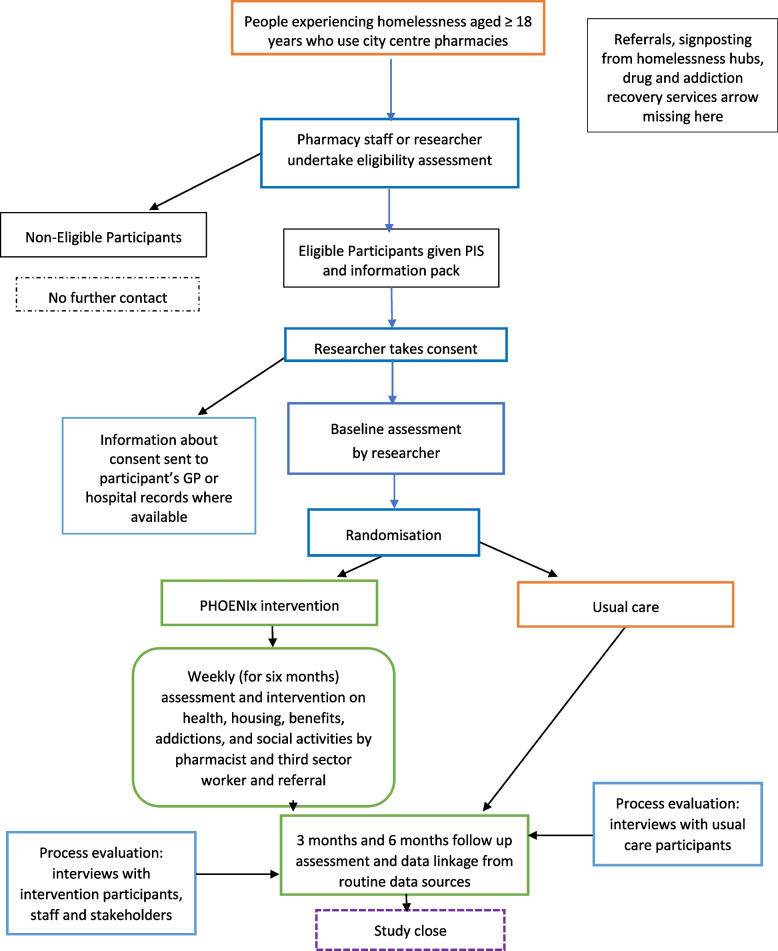
Trial schema

Recruitment and randomisation processes are illustrated in Fig. 1. PEH who attend or who are users of participating Glasgow and Birmingham community pharmacies will be approached by their immediate care team. These include community pharmacists, drug and alcohol recovery services, accommodation providers and a homelessness support hub and addictions team. They will signpost eligible persons to the researcher who can offer potential participants the study information. In addition, community pharmacy staff who know the patient to be experiencing homelessness, can pass study information to them via an invitation pack, explain the study to them and refer any potentially eligible persons to the researchers. Posters and flyers advertising the study will be displayed in pharmacies, homeless charities, drug and alcohol services and temporary accommodations. The study researcher, as delegated on the site signature and delegation log, will confirm study eligibility with potential participants after they have been signposted to the research team via the direct (immediate) care team. Model participant information sheet and consent form are available as Electronic Supplementary Material 1 and Electronic Supplementary Material 2.

Researchers will adhere to The Mental Capacity Act 2005 (England) and Sect. 51 of the Adults with Incapacity (Scotland) Act 2000 in identifying and excluding those individuals who may lack the capacity to consent for themselves or who are under the influence of illicit substances, in recruiting for this pilot trial. The researcher will assess the capacity to consent eligible persons by considering their ability to understand the information relevant to the decision, retain the information and use or weigh the information and communicate his or her decision (by any means). If a patient has managed to attend the pharmacy for collection of medicines and answers to the community pharmacist when asked to present with their name and housing status (the norm during a transaction in the pharmacy), then this will suggest capacity is intact. This is an assessment that community pharmacists are used to making in their daily interactions with this population who tend to be their regular customers.

### Randomisation

The researcher will undertake informed consent followed by baseline data collection. The researcher will then call a dedicated randomisation telephone line at the Birmingham Clinical Trials Unit. Participants will be randomised at the level of the individual in a 1:1 ratio to either intervention or usual care. The randomisation list will be generated by an independent statistician at Birmingham Clinical Trials Unit (BCTU) and will stratify by recruiting city (Glasgow or Birmingham), using permuted blocks of varying lengths. The participant will be informed of the treatment allocation immediately after baseline assessment.

### Blinding

It will not be possible to blind the participant or pharmacist/third sector worker due to the nature of the intervention. It will also not be possible to blind researchers as they will give the participant their allocation (intervention or usual care) and the same researchers will be following up with participants at 3 and 6 months.

### Intervention

The PHOENIx intervention will involve a structured assessment of the participant health and social care status. The prescribing pharmacist will assess physical, mental and problem drug use which includes any relevant near patient tests and/or clinical examination. This will include the provision of harm reduction interventions including injecting equipment provision, foils, naloxone, referral for dry blood spot test for blood-borne viruses. The third sector homelessness support worker will address housing, benefits, advocacy and social prescribing.

Following the initial consultation, the subsequent course of actions are agreed upon between the intervention team and study participants to identify priorities for action. Actions arising from the consultation are implemented over the subsequent weeks during face-to-face consultations. All prescribing activities by the intervention pharmacist follow established clinical guidelines from National Institute for Health and Care Excellence (NICE), Scottish Intercollegiate Guidelines Network (SIGN) or local clinical guidelines or professional societies in accordance with the pharmacists’ professional and clinical judgement. Where necessary, referrals including social prescribing will be made to other services using immediate referral pathways for conditions requiring specialist input, e.g. for mental health crisis presentations. Any changes in the planned intervention will be approved by the ethics committee and recorded in the protocol amendment form.

### Control group (usual care)

The researcher will signpost or refer the participants to other services should they identify urgent health care needs e.g. overdose, or likely infected venous leg ulcer needing dressing, during assessment. In both intervention and usual care groups, participants continue to obtain and seek care, treatment or help as usual.

PEH in the UK are entitled to have registration with a mainstream general practice in their local area. Most urban areas in the UK including both study sites also have provisions for specialist primary healthcare services for PEH. In England, such services are often led by the GP and can offer general medical practice in addition to services for alcohol misuse, physiotherapy and street outreach work. Most of the treatment for other addiction-related services are offered through third party NHS contractors such as ‘Change, Grow, Live’ in Birmingham. In Scotland, PEH are offered a temporary single room in a designated city centre venue, e.g. hotel, hostel or bed and breakfast accommodation, and allocated a named case worker. Patients with problem alcohol or substance use may receive care and treatment from Alcohol and Drug Recovery Service (ADRS) or the Homeless Addictions Team (HAT) or the Heroin Assisted Treatment service. Patients can present to any ADRS seeking help and receive same day assessment. In some circumstances following management of any immediate care needs they may be supported to engage with another ADRS closer to their temporary accommodation or to which they remain open from a previous treatment episode. If transport to a different base is required at the time they present the service will offer a taxi to facilitate this journey. For people already open to ADRS, their care and treatment are provided through a combination of phone and face-to-face contact either at the base or on outreach, dependent on individual needs and circumstances. To access primary health care including a General Practitioner, or ADRS, PEH must either travel to their registered mainstream or specialist homelessness General Practice or phone, which requires PEH to have access to a mobile phone (which can be supplied by ADRS or HAT) or use a landline within their accommodation, if appropriate. All mainstream services operated triage during the COVID-19 lockdown, with requests for patients to phone the relevant care team prior to presenting at the premises, if possible. The Homeless Health Service GP practice offered phone inreach to a variety of homeless accommodation services and restarted outreach after a period of interruption. For patients with mental health problems without problem drug use, access is through a General Practitioner referral to general mental health services or a request for support via ADRS if currently linked in for treatment of problem substance use.

The Pharmacists in the PHOENIx team obtained permission to remotely access all possible health and social care records on outreach, to understand all of the patients’ previous health and social care history and relieve patients of the burden of repeating their traumatic stories again, and for safety reasons. Both intervention and control groups will have the same assessments.

### Incentives

After each researcher visit, the participant will receive a £10 shopping voucher as a token of appreciation for their participation.

### Location

The intervention team will offer consultations mainly in the pharmacy’s private consultation room. Based on the participant’s preferences and ability to visit pharmacies, the intervention team can also arrange to meet study participants in outreach venues at temporary accommodations, homeless hubs or community centres. Where necessary, the intervention team will aim to maximise the uptake of health and social care interventions through assertive outreach assisted by the third sector charity worker to find participants in case of non-attendance at the pharmacy or scheduled venues.

### Withdrawal

Participants will be made aware by the researcher that they can freely withdraw (cease to participate) from the trial at any time. Capacity of the participant to continue in the trial will be assessed by the study researcher during follow-up for the duration of the participant’s time in the trial. If a participant is assessed to have permanently lost capacity, then they will be withdrawn from the trial. Data collected until withdrawal will contribute to outcome assessment.

### Clinical governance

The support offered by the PHOENIx team is fully integrated with existing NHS care providers. The PHOENIx team are connected through remote access, to the respective specialist homelessness health services, general practices and ADRS and hospital records, to enable co-ordinated care. Governance arrangements with the respective GP practices will enable shared clinical records, participation in monthly clinical meetings and checking in with GPs for clinical queries ensuring clinical oversight and safe prescribing.

### Primary outcome measures

The randomisation process and recruitment rate will be measured by the numbers invited to participate, and numbers recruited at baseline. In addition, the retention of those remaining in the trial at 3 months and 6 months follow-up will be measured. Adherence to the intervention will be measured by the proportion of participants attending the intervention at planned visits. The proportion of participants with patient-reported outcomes will be measured by the number of participants who complete assessments at planned visits. Finally, the proportion of participants with routinely collected Emergency Department visits and mortality data available at 6 months will be measured and reported.

### Secondary outcome measures

Secondary outcome measures have been divided into clinical measures, addiction-related measures and social outcome measures. These are listed in Table 1.

**Table 1:** Secondary outcome measures

Clinical outcome measures	Addiction measures	Social measures
1. Number and cause of Emergency Department visits measured by patient-reported and routinely collected data at 3 and 6 months 2. Number and cause of primary care general practice visits measured by patient-reported and routinely collected data at 3 and 6 months 3. Mortality measured by routinely collected data at 6 months 4. Medication changes (prescribed) and taken (in the case of opioid substitution therapy where supervised) measured through patient-reported and routinely collected data at 3 and 6 months 5. Number and cause of hospitalisation measured by patient-reported and routinely collected data at 3 and 6 months 6. Intervention acceptability measured by qualitative interviews with patients, the intervention delivery team and stakeholders as an ongoing process evaluation 7. Generic health-related quality of life score and health thermometer score measured by EQ-5D-5L at 3 months and 6 months 8. Frailty measured by Fried’s adapted frailty phenotype at 3 months and 6 months 9. Respiratory health measured by Peak Expiratory Flow Rate, MRC Dyspnoea scale and COPD Assessment Test (CAT) at 3 months and 6 months 10. Blood pressure measured by blood pressure monitors at 3 months and 6 months	1. Number of participants experiencing drug overdoses not requiring an Emergency Department visit and the number of overdoses measured by patient-reported data at 3 months and 6 months 2. Number of participants (and number of times) referred to drug and alcohol services, rehab, mental health and GP, and the numbers attending subsequently measured by patient-reported and routinely collected data at 3 and 6 months 3. Numbers and time to commencement on opiate substitute treatment (OST)/benzodiazepine/heroin-assisted treatment and collecting ≥ 80% of daily doses measured by routinely collected data at 3 months and 6 months 4. Number of participants treated with and time to minimum therapeutic OST dose measured by routinely collected data at 3 months and 6 months 5. Dose of OST measured by routinely collected data at 3 months and 6 months 6. Number of missed appointments (with any team, including irregular discharges) and number of participants with missed appointments measured by routinely collected data at 3 months and 6 months 7. Number of persons and days in prison/criminal justice encounters measured by patient-reported data at 3 and 6 months	1. Housing tenure including night shelters, emergency accommodation measured by self-reported data and those provided by the council or third sector, or care home at 3 months and 6 months 2. Level of debt as self-reported by patients at 3 months and 6 months 3. Criminal justice encounters as self-reported by patients at 3 months and 6 months

### Fidelity testing

The qualitative researcher will check the information entered onto a randomly selected subset of participants’ care plan/clinical records by the pharmacist by reviewing anonymised clinical care records kept by the PHOENIx team and transferred to the qualitative team. The qualitative researchers will then compare these data against a checklist of the components of the intervention: physical health; mental health; addictions; housing; debt; and social activities. This will be used to provide iterative feedback to the intervention team to ensure fidelity and optimise the delivery of the intervention, and in addition, will address intervention fidelity.

### Process evaluation

Semi-structured interviews with a purposive sample of 15–20 participants in the intervention group, 7–10 health professionals and approximately 10 stakeholders including commissioners/senior health board/homelessness services policy makers and representatives from volunteer sectors will be undertaken in order to explore their views on the trial methods and barriers and facilitators to future implementability. We will use Normalisation Process theory [24] to help us conceptualise the effects of the intervention on the interplay between patient capacity and self-management workload which will help us refine our preliminary logic models (which will be developed early in the pilot study) in relation to the intervention. Interviews will be audiotaped with participant consent and transcribed to provide data for qualitative analysis.

### Statistical analyses

#### Quantitative data

Feasibility measures will be analysed using appropriate summary measures including proportions, along with 95% confidence intervals (based on a normal approximation method for one sample proportions) to describe uncertainty. For clinical measures, the study sample size is too small to allow a reliable analysis of the effect of the intervention on outcomes. No hypothesis testing will be performed and the analysis will be limited to estimates of effect size and measures of uncertainty where appropriate. The primary comparison groups will be composed of those randomised to the trial intervention versus those randomised to the control group (usual care). In the first instance, all analyses will be based on the intention to treat principle, i.e. all participants will be analysed in the intervention group to which they were randomised irrespective of adherence to randomised intervention or protocol deviations. Appropriate summary statistics and differences between groups (e.g. mean differences, relative risks, absolute differences) will be presented, with 95% confidence intervals. A log-binomial regression model will be used for binary outcomes and a linear regression model used for continuous outcomes, adjusting for recruiting city and baseline response (where applicable).

### Process evaluation and associated outcomes

#### Qualitative data

Interview transcripts will be analysed using a framework approach underpinned by Normalisation Process Theory, an implementation theory with four constructs: coherence (sense making); cognitive participation (engagement); collective actions (operationalisation) and reflexive monitoring (appraisal) [24, 25].

### Economic evaluation

Resource use data will be identified and measured with unit costs applied (from routine sources where possible) to value all available health, social care and personal resource use data. The responses will cover the number of people incurring each resource and the number of times each resource was incurred by each person who experienced it. The possibility of an evaluation incorporating a wider societal (or at least public service perspective) in line with the work already conducted, will be considered to establish broader costs and benefits beyond the NHS. The main measure of benefit explored will be the EQ-5D-5L [26], cross-walked to the EQ-5D-3L using the method recommended by NICE by default [27, 28] and consider the impact of using the EQ-5D-5L value set. If unanticipated issues arise in data collection that leads to it not being possible to use both the EQ-5D-5L data or the resource use data, the results of the economics work would simply summarise narratively where there have been successes in data collection for economic-relevant outcomes, compared to where this was less successful, in order to inform methods of data collection that are more likely to facilitate good response rates amongst PEH for future RCTs in this area.

### Trial oversight committee

Oversight of the PHOENIx trial will be provided by an independent Trial Oversight Committee (TOC). The Committee will meet via teleconference/face-to-face approximately twice a year or as required depending on the needs of the trial. The TOC will provide overall oversight of the trial, including the practical aspects of the trial, as well as ensuring that the trial is run in a way which is both safe for the participants and provides appropriate feasibility data to the sponsor and investigators. An independent data monitoring committee will not be set up for this trial and the role will be fulfilled by the Oversight Committee. The intervention is considered low risk. An emergency meeting may however be convened if a safety issue is identified.

### Progression criteria

The aforementioned primary and secondary outcomes along with progression criteria will be considered by the TOC to determine whether the study should proceed to a subsequent definitive trial (Table 2):

**Table 2:** Progression criteria

	**Red (discuss with Oversight Committee and consider substantial changes before proceeding to the definitive trial)**	**Amber (discuss with Oversight Committee strategies for improvement and consider changes to processes before deciding whether to proceed to full trial)**	**Green (go ahead)**
**Recruitment**			
Proportion of PEH (as assessed by the researchers) meeting eligibility criteria and agreeing to participate	< 40%	40–50%	> 50%
**Retention**			
Proportion of participants remaining in the study at 6 months	< 50%	50–60%	> 60%
**Intervention adherence**			
Proportion of participants attending > 50% of intervention visits as planned (flexible schedule agreed at consultation)	< 50%	50–60%	> 60%
**Outcome data**			
Proportion of participants with Emergency Department visits and mortality data available at 6 months	< 60%	60–70%	> 70%
Proportion of patients with questionnaire booklets completed at 6 months	< 50%	50–60%	> 60%

*PEH* persons experiencing homelessness

The data collected in this pilot study will inform the required sample size for the future definitive trial. It is likely that the primary outcome for the definitive RCT to be a composite outcome of mortality and Emergency Department visits.

### Data management

Data entry will be completed by the sites on paper and sent to Birmingham Clinical Trials Unit to be entered. The data capture system will conduct automatic range checks for specific data values to ensure high levels of data quality. Queries will be raised using data clarification forms via the trial database. The sponsor has policies in place, which are designed to protect the security, accuracy, integrity and confidentiality of Personal Data. The trial will be registered with the Data Protection Officer at Birmingham. Data will be held in accordance with the Data Protection Act (2018 and subsequent amendments). The Trial Office has arrangements in place for the secure storage and processing of the trial data which comply with data security policies at the relevant institution.

## Discussion

As far as we are aware, no published or unpublished RCTs have considered pharmacist-led support for PEH in a community pharmacy setting integrated with a third sector support worker. This study aims to address this gap. The inclusion of clinical pharmacists in the primary care team is embedded in NHS primary care framework and practice models in the UK [29, 30]. These have been shown to improve: appointment access; medication adherence; patients’ understanding of long-term conditions; minimising illicit opioid use; and establishment of good quality networks with community pharmacy [31]. Approximately 7500 (of 56,000) pharmacists in the UK are qualified independent prescribers allowing them to diagnose medical conditions, prescribe medicines and make referrals. While many pharmacists in general practices and the PHOENIx team (within low threshold venues including homelessness drop in centres and emergency accommodation), have been undertaking prescribing roles, and have RCT evidence of impact [32], currently there is a lack of opportunities in community pharmacy for PEH to access care and pharmacist independent prescribers to utilise their skills. The involvement of a third sector homelessness worker collaborating fully with NHS prescribing pharmacists in Community Pharmacies also offers a new approach to care delivery for PEH who struggle to access care.

The proposed study also aligns well with the national and international policies focused on minimising health disparities. These policies underscore the need for innovation in health care delivery for PEH and many of the principles are part of the PHOENIx intervention. The proposed PHOENIx model supports current strategic priorities for the inclusion of health and health equity in the NHS and local government levels [33, 34] and the crucial role community pharmacy has to play in promoting health, well-being and prevention, given their broad expertise, accessibility and knowledge of their communities.

Evidence from this study will be used to inform an NHS-funded free-to-access service for PEH in community pharmacy. The findings of this study will be shared with the study funder, National Institute for Health and Care Research. The report will also be available on the study website. We plan to share the pilot study results with relevant stakeholders with the aim of further engagement and support for the main study. We will also present and share our findings directly with patients in pharmacies, accommodation sites, and low-threshold venues in Glasgow and Birmingham. Our research team represent local and national constituents and stakeholders in addictions, recovery groups, criminal justice, homelessness, general practice, academia and pharmacy. Our charity co-applicant partners will disseminate in their networks. It is anticipated that all co-investigators including trial team members from the Birmingham Clinical Trials Unit will contribute to the writing and editing of manuscripts for publications resulting from the trial and fulfil the International Committee of Medical Journal Editors criteria for authorship.

### Study limitations

Given the pilot nature of this study, the study will not be powered to meaningfully evaluate the differences in clinical or social outcome measures. The study will primarily rely on PEH walking to pharmacies. Given their health and inability to commute, many PEH would be disadvantaged by solely using this primary mode of recruitment. However, study researchers will ensure wider users of pharmacy including those receiving prescription through delivery services in their accommodations and/or through any other remote/online services will also be identified and recruited. The study will be conducted in two cities within the UK which share the important burden of deprivation. Patterns of homelessness and health problems, however, may vary across settings. In addition, social circumstances also rely on support and governance in local city authorities. Bias is possible, although unavoidable, through signposting the usual care group for urgent support measures such as referral to Emergency Department or to other health and social care staff.

## Reporting

Standard reporting checklist is appended with this manuscript as Electronic Supplementary Material 5.

## Supplementary Information

**Additional file 1.** PHOENIx Consent form.

**Additional file 2.** PHOENIx PIS.

**Additional file 3.** Peer review reports.

**Additional file 4.** Peer review reports.

**Additional file 5.** SPIRIT.

## Data Availability

Only scientifically sound proposals from appropriately qualified Research Groups will be considered for data sharing. The request will be reviewed by the BCTU Data Sharing Committee in discussion with the Chief Investigators and, where appropriate and where appropriate with any of the following: the Trial Sponsor, the relevant Trial Management Group and independent Trial Oversight Committee.

## Abbreviations

ADRS: Alcohol and Drug Recovery Service
B&Bs: Bed and breakfast
BCTU: Birmingham Clinical Trials Unit
CAT: COPD Assessment Test
ED: Emergency Department
ETHOS: European Typology of Homelessness and housing exclusion
GP: General Practices
HAT: Homeless Addictions Team
MRC: Medical Research Council
NHS: National Health Service
NICE: National Institute for Health and Clinical Excellence
OST: Opioid substitution therapy
PEH: Persons experiencing homelessness
PHOENIx: Pharmacy Homeless Outreach Engagement Non-medical Independent prescribing Rx
PPI: Patient and public involvement
QALY: Quality adjusted life year
RCT: Randomised controlled trial
SIGN: Scottish Intercollegiate Guidelines Network
