# Supplementary material for: Protocol for a pilot randomised controlled trial to evaluate integrated support from pharmacist independent prescriber and third sector worker for people experiencing homelessness: the PHOENIx community pharmacy study
Source: Pilot Feasibility Stud. 2023 Feb 23;9:29. doi: 10.1186/s40814-023-01261-x (PMC9946705; doi:10.1186/s40814-023-01261-x)
Supplement: Supplementary file 1 — Additional file 1. PHOENIx Consent form. [file 40814_2023_1261_MOESM1_ESM.docx]

**P**harmacy **H**omeless **O**utreach **E**ngagement **N**on-medical **I**ndependent prescribing R**x** (**PHOENIx**) community pharmacy-based pilot randomised controlled trial

**Consent Form**

Participant ID:

**CONFIDENTIAL ONCE COMPLETED**

**Please INITIAL or THUMBPRINT each box to indicate that you agree with the statement:**

| I confirm that I have read and understood the **PHOENIx** Community Pharmacy information sheet dated DD-MMM-YYYY (Version X.X) for the above study. I have had the opportunity to consider the information, ask questions and have had these answered to my satisfaction. |  |
| --- | --- |
| I understand that my participation is voluntary and that, if I take part in the **PHOENIx** Community Pharmacy trial, I may withdraw at any time without having to give a reason. I am aware that this will not affect my medical care or legal rights. I understand that even if I withdraw, data collected up to my time of withdrawal, may still be used for the **PHOENIx** Community Pharmacy trial. |  |
| I understand that data will be collected by my local research team, including a copy of my consent form which identifies me by name, as well as other personal information (address, date of birth, and telephone number). I understand this information will only be used for the purposes of the research and that it will be transferred to University of Birmingham or University of Glasgow, stored securely and treated confidentially. I agree to the transfer and storage of this data. |  |
| I understand that I may be contacted by researchers to take part in an interview to ask questions about my health, how I feel and also about my views of the study. I understand the interview will be audio-recorded and the file stored securely.  I understand anonymised quotes from the interview may be used in publications. |  |
| I understand that relevant sections of my medical notes and data may be looked at by individuals from the University of Birmingham, University of Glasgow and the **PHOENIx** Community Pharmacy research team, where it is relevant to my taking part in this research. University of Birmingham may request NHS organisations, Ambulance Service, Prisons health services NHS data services, NHS service contractors, SIFA Fireside, Simon Community Scotland or temporary accommodation providers to access and collect information about my health and social/housing status using my identifiable information. I give permissions for these individuals and organisations to have access to these records and share data with the PHOENIx research team and contact me using the relevant information where needed. |  |
| I understand that relevant information (as detailed in the patient information sheet) held by Simon Community, SIFA Fireside, Prison services or temporary accommodation providers may be used or looked at by individuals from the University of Birmingham, University of Glasgow and the **PHOENIx** Community Pharmacy research team, where it is relevant to my taking part in this research. I give permission for these individuals to access this information. |  |
| I give permission (where applicable) for my GP to be informed of my participation in the **PHOENIx** Community pharmacy study. I understand that the pharmacist will discuss any relevant changes to my medicines with my GP and any other relevant healthcare professionals. |  |
| I understand that whether I am in the usual care or the **PHOENIx** Community pharmacy group, the researcher will visit me in the pharmacy, a homeless charity hub or where I stay, at baseline and at approximately 3 months and 6 months. |  |
| I understand that the data collected during the study will be pseudonymised, used for medical research and teaching only and that I will not be identified in any way in the analysis and reporting of the results. I understand that the information collected about me will be used to support other research in the future, and may be shared anonymously with other researchers in future studies/for secondary analysis.. |  |
| I understand that data collected during the study will be archived for regulatory bodies auditing the conduct of this research and who may also have access to this anonymised information, for up to 10 years after the study has finished. I understand that this information will be stored securely and treated confidentially. I give permission for individuals from regulatory bodies to have access to this information. |  |
| I agree for my contact details to be used to send me a summary of the study findings. |  |
| I agree to take part in the **PHOENIx** community pharmacy study. |  |

Name of Participant (print) Date Participant Signature (or thumbprint in box)

____________________________ DD/MMM/YYYY _________________________________

Name of Witness (if applicable) Date Witness Signature

____________________________ DD/MMM/YYYY _________________________________

Name of Person Taking Consent (print) Date Signature

____________________________ DD/MMM/YYYY _________________________________

**When completed: Original to be kept in Investigator Site File, one for GP, one for outreach worker, one copy for Glasgow and one copy posted to BCTU**
