# Supplementary material for: Protocol for a pilot randomised controlled trial to evaluate integrated support from pharmacist independent prescriber and third sector worker for people experiencing homelessness: the PHOENIx community pharmacy study
Source: Pilot Feasibility Stud. 2023 Feb 23;9:29. doi: 10.1186/s40814-023-01261-x (PMC9946705; doi:10.1186/s40814-023-01261-x)
Supplement: Supplementary file 2 — Additional file 2. PHOENIx PIS. [file 40814_2023_1261_MOESM2_ESM.docx]

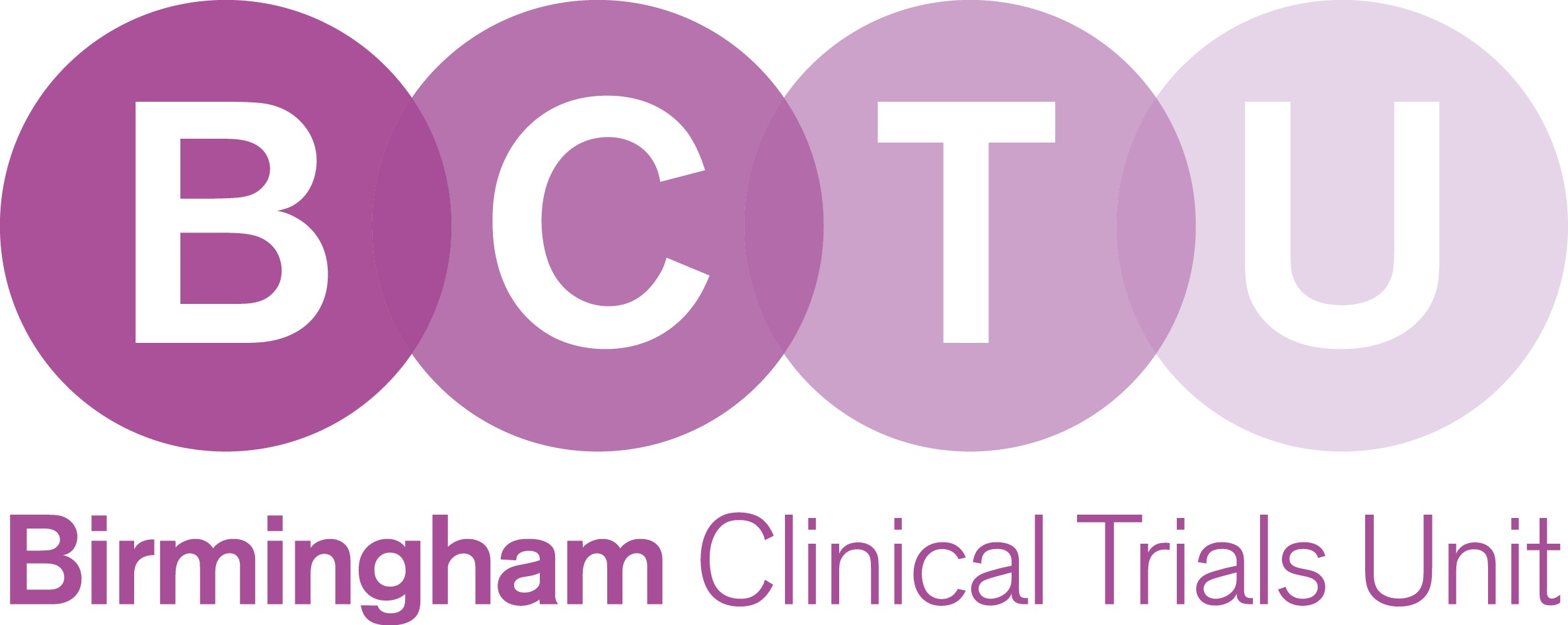

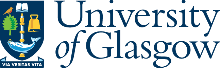

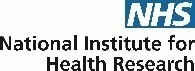

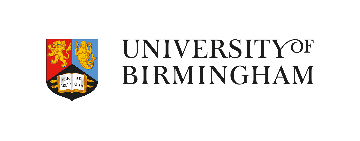


Participant Information Sheet


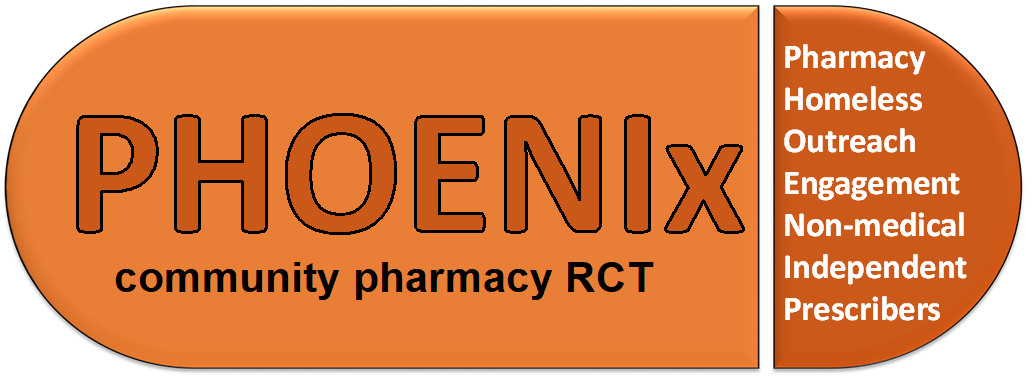

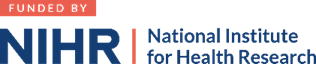

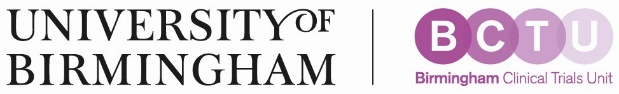


**You are invited to take part in the PHOENIx Community Pharmacy Study.**

Before you make your decision, it is important for you to understand why the research is being done and what it will involve.

Please take time to read the information in this leaflet. You may want to talk to others about the study before deciding to take part.

PIS Feasibility PIS v0.1 date **08/02/2021** IRAS: ISRCTN

# Pharmacy Homeless Outreach Engagement Non-medical Independent prescribing Rx (PHOENIx) community pharmacy-based pilot randomised controlled trial

Summary

This study is for people who are currently experiencing homelessness.

We want to find out if we can improve the health of people who are experiencing homelessness.

If you were to take part in the study, you would be randomly allocated to one of two groups. One group would receive treatment and support from a pharmacist and a third sector homeless support worker with weekly visits for up to 6 months, in addition to usual services. The other group would receive signposting to usual services (primary care services such as your GP or other support service).

In both groups a researcher will contact you at 3 and 6 months after you join to follow up with you. You may be invited to take part in an interview about your experiences.

# Why are you doing this project?.

We know that you are going through a difficult time in your life, so the PHOENIx team want to see if we can help improve your health by adding to any existing support (such as your GP or addictions services) that you have in place.

The intervention we are testing is additional, weekly support from a NHS Pharmacist and a homelessness support worker (the PHOENIx team) who will be joined by a researcher during most appointments.

The findings from this study will help us identify how well (if at all) the PHOENIx team help you. The findings will tell us if we should plan a larger study to see if the PHOENIx team can improve the health of other people who are experiencing homelessness.

# Why have I been invited to take part?

You are being asked to take part because you are currently experiencing homelessness and are over 18 years old.

# What would taking part involve?

Taking part in the study will last for **6 months**.

If you take part, you will be randomly allocated to either:

- Intervention group: You will receive weekly visits from a pharmacist and the third sector homelessness support worker for up to six months
- Control group: be signposted to your primary care services such as your GP or other support service

Whichever group you are allocated to you will be asked to do the following:

1.
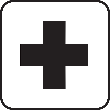
Provide some medical history, demographic information (e.g. age, ethnicity) and contact details.
2.
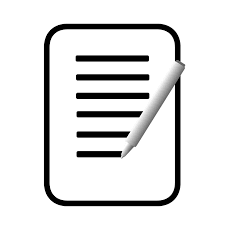
Complete a set of **questionnaires** at 3 different time points: **Baseline, 3 months** and **6 months** into the study. These contain questions about your health including physical health, mental health fatigue (tiredness), quality of life, addiction, medication and healthcare/ support as well as housing tenure, debt and criminal justice encounters. The questionnaires take about 30 minutes to complete.
3.
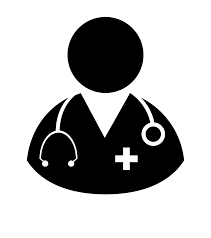
Our researcher will conduct 6 quick checks:

1. Breathing (exhale into a hand held machine).

2. Pulse oximetry (small device strapped onto your finger tip for 10 seconds to check blood oxygen level).

3. Temperature (using ear temperature probe).

4. Weight and Height

5. Grip strength (this will involve squeezing a machine that tests your hand grip strength).

6. Blood pressure

And if needed, and if you agree, make a referral to your primary care or addiction services for a dry blood spot test for Hepatitis C virus and Human Immunodeficiency Virus (HIV)- if you have not had one in the past 3 months.

1.
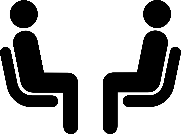
A handful of participants from both groups will be invited to attend an **interview** with a researcher to talk about their experience of taking part in the study.

The interview will last between 30-60 minutes. The researcher will ask you questions about your experiences of taking part in the PHOENIx study, including:

- Completing questionnaires
- Your experiences of entering the study
- The intervention (if applicable)
- Positive and negative impacts

The interview will be recorded using an audio recorder, this helps us remember what has been discussed.

You can choose where you would like the interview to take place; for example, it could be at the pharmacy where you joined the study or another place you feel comfortable. We could also do the interview by phone if that is an option for you.

If you agree to take part in the study, we will take your informed consent to participate in this research. This will involve you signing a Consent Form. You will have the opportunity to ask questions about the study and time to consider your participation. If you have trouble reading, the researcher will read this leaflet and the consent form to you for you to initial or thumbprint.

# What happens if I am allocated to the Intervention group?

If you are in the intervention group, the pharmacist and the third sector homelessness support worker will assess your needs then work with you to address them. Together you will be offered weekly visits (where possible) in the pharmacy, at your place or other venues such as a homelessness hub. You will have access to this level of support for six months. We (the research team) may attempt various ways to contact you if you do not attend the scheduled appointment for any reason. This may include the research team contacting you on the phone number you have provided, or liaising with services such as your temporary accommodation providers/city centre homelessness venues as part of their method to contact you.

# What happens if I am allocated to the Control group?

If you are in the Control group, the pharmacist will assess your needs then signpost you to relevant support agencies such as your primary care (e.g. GP) or alcohol and drug support services.

# How would I be allocated to the Intervention or Control group?

Once you have provided your consent to be in the trial, the researcher will note down some basic information about you. We (the research team) will then need to speak to the University of Birmingham who will provide the researcher with the next allocation on the list. The allocation list is random, like tossing a coin, so that neither the researchers nor the pharmacist can know which allocation is coming next.

Questions you might want to ask

# Do I have to take part?

No. Taking part is totally voluntary. If you change your mind after you’ve consented, you may withdraw from the study, or parts of the study, at any time. To do this, just let the researcher or the study team at the University of Birmingham know. Contact details can be found further on in this leaflet.

If you do withdraw from the study, any data collected from you up until the point of withdrawal will be kept.

# What data will you be collecting?

We will ask for your consent before collecting the data below. This is only for use in the study and we will try to make this as simple as possible.

| **When** | **What data will be collected** |
| --- | --- |
| **When you agree to take part** | - Name, date of birth and NHS/Community Health Index (CHI) number - Contact details: phone number, email, housing status - Sex, ethnicity, working status - Physical and mental health conditions smoking status, alcohol consumption, height, weight, blood pressure, cholesterol (most recent record), hand grip strength - Drug use, number of emergency department visits in last 6 months - Current medications you are taking - Disability status - Benefits status, social activities - We will also collect the information listed below for 3 and 6 months at this appointment |
| **Baseline, 3 and 6 month Questionnaires** | Questionnaires contain questions regarding:   - Health related quality of life - Anxiety/ depression - Stage of Chronic Obstructive Pulmonary Disease (COPD)- a respiratory disease - If medication is being taken as prescribed - Frailty - Use of healthcare and support services - Emergency department visits - General Practice visits - Quality of Life - Drug overdoses (not requiring hospitalisation) - Referrals to Addiction and Drug Recovery Service (ADRS) or addictions services, rehab, mental health and primary care, and numbers attending subsequently - Commencement on Opioid substitution therapy/Benzodiazepine/heroin assisted treatment - Treatment retention - Opioid substitution therapy - Missed appointments - Housing tenure - Level of debt - Time in rehabilitation - Criminal justice encounters - Time in prison |
| **Data from health and other registries** | - Emergency Department visits, in-patient hospital stays, criminal justice encounters, Ambulance service call outs, alcohol and drug recovery services, complex needs and homelessness outreach services |

# Will I be paid to take part?

We will provide you with a £10 shopping voucher provided at baseline and at the 3 and 6 month follow up appointment.

# What are the possible benefits of taking part?

In the future, this could help improve care for people who are experiencing homelessness.

If you are allocated to the intervention group you will receive additional follow-up care, which you may not normally receive, to help identify and address your needs.

Information we get from this study will help us plan a larger study to test how effective the PHOENIx intervention is, and whether it improves quality of life.

# What are the possible disadvantages and risks of taking part?

Based on a small previous study we have done, we do not anticipate any disadvantages if you decide to take part. You will need to give your time to participate in the study. If you are in the intervention group you will be offered visits with the pharmacist and the third sector homelessness support worker (Simon Community/SIFA worker) on a schedule that suits you, over a maximum of 6 months. We will require participants in both groups to complete questionnaires when they join the study and at three and six months.

There is a potential that the interview may be tiring or the topics we talk about may be challenging. However, you will be able to take a break or stop the interview at any point. If the researcher becomes concerned for your wellbeing during the interview, they will discuss their concerns with you and work in partnership with you to determine the best course of action. If necessary, the researcher may contact your primary care service such as your GP if they are concerned for your wellbeing. Taking part in the study and any answers you give in questionnaires or interviews will not affect your treatment in the NHS, your care or your legal rights.

# Will you involve my General Practitioner (GP)?

With your permission, your GP will be informed of your participation in the study. If you are in the intervention group, your GP will be sent a copy of your action plan and a summary of the follow-up appointment. If you are not registered with a GP, you will be given the opportunity to register with the homelessness GP service.

# What will happen to the results of the study?

A summary of the results will be available on the study’s website www.birmingham.ac.uk/PHOENIx. You will be given the option to be provided with a summary of the results if you take part in the study. The results will also be published in a scientific journal and presented at scientific conferences. You will not be identified in any of the reports or publications.

# Will my information be kept confidential?

We will only use information about you that we need for the research study. We would like to request information about you from other organisations to help us collect data to see how taking part in this study has impacted your health and wellbeing. These organisations include Simon Community, SIFA Fireside, UK NHS bodies such as NHS Digital NRS Scotland, other NHS data portals ambulance services, temporary accommodation providers and prisons healthcare. Information held and maintained by these organisations may be used to help contact you or provide information about your health and social/housing status. Your identifiable information held and maintained by the University of Birmingham, University of Glasgow or NHS Glasgow & Clyde may be sent to these organisations or database portals to link your information to the data these organisations hold and maintain about you for the purpose of the PHOENIx study to enable us to obtain the information about your health and any impact that PHOENIx intervention may have on your health The research team will have data protection arrangements through data sharing agreement where relevant

If you take part in the interview, the recording of the interview will be typed up but will be pseudonymised (this means that all names and other identifiable information will be removed and your data will be linked to a unique ID number that will allow only those with permission to know that the data belongs to you). We will transfer the recording onto a secure computer at the University of Glasgow and then we will delete from the recorder. A professional transcription company will type up the recording, which will be transferred to the transcription company through a secure file transferring website. A confidentiality agreement will be in place with the transcription company. We will delete the recordings once we have checked the transcription. In the transcription, your identifiers (e.g. names and addresses) will be removed and pseudoanonymised with a study number. Any information that identifies any other person will also be anonymised.

Everyone involved in this study will keep your data safe and secure. We will also follow all privacy rules. We will make sure that no identifiable data about you is shared in publications, conference or any outputs from this study. We will anonymise any quotes from the interviews before being used in any publications. The Data Protection Information section tells you more about this.

From time to time we may be asked to share the study information (data) we have collected with researchers running other studies in this organisation and in other organisations so that they can perform analysis on the data to answer other important questions. These organisations may be universities, NHS organisations or companies involved in health research in the UK. Any such request is carefully considered by the study researchers and will only be granted if the necessary procedures and approvals are in place. This information will not identify you and will not be combined with other information in a way that could identify you. The information will only be used for the purpose of health research, and cannot be used to contact you or to affect your care. It will not be used to make decisions about future services available to you, such as insurance. Under no circumstances will you be identified in any way in any report, presentation or publication arising from this or any other study.

# Are there any circumstances when my confidentiality will be broken?

Please be aware that the researcher has a duty to inform appropriate authorities if you disclose information that either indicates a risk or harm to yourself or others.

# What will happen if I don't want to carry on with the study?

You can stop being part of the study at any time, without giving a reason, but we will keep and process information about you that we already have.

We need to manage your records in specific ways for the research to be reliable. This means that we won’t be able to let you see or change the data we hold about you. For information on your rights in relation to your data, please see the relevant section in this information sheet. Data collected until withdrawal will still be used.

You are free to withdraw your interview data from the study without giving a reason up to 5 days after the interview. This is because after 5 days the data will be integrated into our data set. If you withdraw your data, it will not affect your current or future healthcare or legal rights. If you would like to withdraw your data after the interview please contact the researcher using the contact details at the end of this booklet.

# What will happen after the trial?

After the trial, we will use the results to decide if we should conduct a larger clinical trial of the intervention. We may be able to continue the intervention in Glasgow, but may not be able to continue in Birmingham.

# What if new information becomes available?

We will keep you updated with any new information relevant to your participation in the study. If the new information may affect your decision to continue participating in the study, you will be given time to consider your participation and if happy to continue we will re-take your consent.

# What if something goes wrong?

We do not envisage any problems as a result of your participation in the study. However, all patients are covered for negligent harm from clinical treatment according to NHS insurance guidelines. In addition, the study sponsor (the University of Birmingham) provides cover for harm which comes about through the University’s, or its staff’s, negligence in relation to the design or management of the trial .lIf you have a concern about any aspect of this study, you should ask to speak to a member of the research team who will do their best to answer your questions.

If you wish to complain about any aspect of the way you have been approached or treated during the course of this study, the normal National Health Service complaints mechanisms will be available to you. Copies of these guidelines are available on request. If you wish to complain about how you have been treated during this study please contact Patient Advice and Liaison Service (PALS) at your local hospital. Contact details can be found on the end of this Patient Information Sheet or via this website: <https://www.nhs.uk/common-health-questions/nhs-services-and-treatments/what-is-pals-patient-advice-and-liaison-service/>

# What happens if I lose capacity to consent during the study?

In the event that you have permanently lost capacity to consent during the study, we (the research team) would retain data already collected for the study. We will not collect any further data about yourself.

# How have patients and the public been involved in this study?

We obtained help and advice from a group of individuals experiencing or who have previously experienced homelessness and representatives of that community as well as members of the public to develop this research topic and the research questions that should be asked.

This group were involved in reviewing the documents for this study, including this information sheet. They provided to us the advice on the study design, in particular giving opinions on the frequency of participant visits and the tests that will be carried out. The group continues to be involved in the study and provide valuable insight.

# Who is organising and funding the research?

The study is co-ordinated by Birmingham Clinical Trials Unit and is sponsored and insured by the University of Birmingham (reference: RG_20-123). The Chief Investigator is Dr Vibhu Paudyal and Dr Richard Lowrie is the joint study lead. This study is funded by a National Institute for Health Research (NIHR), HS&DR (ref. 133060).

All research in the NHS is looked at by an independent Research Ethics Committee, to protect your interests. This study is approved by TBC.

# If I want to take part, what happens next?

Please let the researcher who gave you this information sheet know that you would like to take part. We will then start the process of getting your consent to join the study.

**THANK YOU for taking the time to read this information sheet**

If you would like further information or would like to speak to someone about the study please contact:

|  | **Dr Vibhu Paudyal (Chief Investigator)**  **Dr Richard Lowrie (Study Lead)**  ***Email:*** [PHOENIx@trials.bham.ac.uk](mailto:PHOENIx@trials.bham.ac.uk)  **Birmingham**: PHOENIx trial office, Birmingham Clinical Trials Unit, University of Birmingham, Edgbaston, B15 2TT.  **Glasgow:** Homeless Health (PHOENIx) team, Pharmacy Services, NHS Greater Glasgow and Clyde, Glasgow, G76 7AT |
| --- | --- |
| 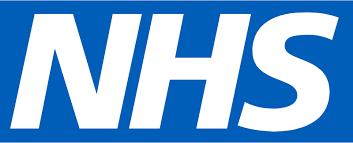 | **<Insert PI name> (Principal Investigator)**  ***Phone:*** <Insert local trust phone number>  ***Address:*** <Insert local trust address> |
| 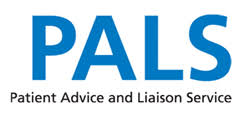 | **Patient advice and liaison services (PALS)**  **Phone:** <insert> **Email:** <insert> |

Data Protection Information

This section is intended to summarise and explain how this research will process your personal data following the Data Protection Act 2018. It seeks to help you understand why it is important and we suggest you read this information carefully.

# How will we use information about you?

We will need to use information from you and your medical records for this research project. This information will include your name, NHS number, contact details, ethnicity, sex, and frailty scores. People will use this information to do the research or to check your records to make sure that the research is being done properly. These people will include the PHOENIx Community Pharmacy research team, individuals from the sponsor, NHS Digital and other regulatory organisations.

People who do not need to know who you are will not be able to see your name or contact details. Your data will have a code number instead. We will keep all information about you safe and secure.

Once we have finished the study, we will keep some of the data so we can check our results. We will write our reports in a way that no-one can work out that you took part in the study.

# Who will my personal data be shared with?

For the purposes of the research project, we will only share your data with third parties required to conduct the research. Sometimes, external organisations assist us with processing your information, for example, a professional transcription company will be used to type up the audio recordings of the interviews. These organisations act on our behalf in accordance with our instructions and do not process your data for any purpose over and above what we have asked them to do. We make sure we have appropriate contracts in place with them to protect and safeguard your data. If your personal data are transferred outside the European Union (for example, if one of our partners is based outside the EU or we use a cloud-based app with servers based outside the EU), we make sure that appropriate safeguards are in place to ensure the confidentiality and security of your personal data.

We will also share your data with NHS Digital who will help us to understand how you have accessed the NHS during your time in the study.

In relation to this project, any paper records will be kept in a locked filing cabinet, in a locked room in a building with controlled access. Any electronic data will be stored securely on University or NHS owned servers that are password protected and access is user defined.

# How long will my personal data be kept?

We will destroy your personal data after analysis. We will retain anonymised research data for 10 years after the publication of the research outcomes to allow it to be verified if necessary. If you withdraw from the project, we will keep the information we have already obtained but, to safeguard your rights, we will use the minimum personally-identifiable information possible.

# Where can you find out more about how your information is used?

You can find out more about how we use your information

- at [www.hra.nhs.uk/information-about-patients/](https://www.hra.nhs.uk/information-about-patients/)
- our leaflet available from [**www.hra.nhs.uk/patientdataandresearch**](http://www.hra.nhs.uk/patientdataandresearch)
- by asking one of the research team
- by sending an email to dataprotection@contacts.bham.ac.uk
