# Supplementary material for: Protocol for a pilot randomised controlled trial to evaluate integrated support from pharmacist independent prescriber and third sector worker for people experiencing homelessness: the PHOENIx community pharmacy study
Source: Pilot Feasibility Stud. 2023 Feb 23;9:29. doi: 10.1186/s40814-023-01261-x (PMC9946705; doi:10.1186/s40814-023-01261-x)
Supplement: Supplementary file 3 — Additional file 3. Peer review reports. [file 40814_2023_1261_MOESM3_ESM.pdf]

NIHR | National Institute for Health Research

NIHR Health Services and Delivery Research Programme

RESEARCH PROPOSAL - EXTERNAL REVIEW OPEN COMMENTS

**Comments**
Research Team<br>- I don’t understand the justification of such a large research team for such a small study. Particularly with many of the applicants listed as <1% FTE and two as 0%FTE.<br>- Justification for joint lead<br>o The applicants need to better explain what they mean by recruiting a ‘wide team’. Do they mean that many people will be recruited; or will they recruit people with a breadth of skills? Either way, the decisions need to be explicitly stated and justified as they no doubt incur a cost.<br>o Unsure how ‘Paudyal collaborates closely with Birmingham CTU’ justifies 15% FTE.<br>- Relevant expertise – would be interested to see a demonstrable track record of influencing national policy and practice.<br><br>Project related comments:<br>- More explicitly stated ‘promising quantitative and qualitative outcomes’ from the feasibility study may make this a more convincing proposal.<br>- The study presumes that PEH will happily walk in and disclose a great deal of health and other personal information to someone they have just met. It also assumes a high degree of trust in pharmacists.<br>- It seems that many PEH will be signposted to other services, need to more explicitly state what the role is that pharmacists will play. Giving practical examples may be helpful.<br>- People living in shelters and temporary accommodation very often have access to support and signposting through these services; their inclusion in the study thus seems to double up work. The proposal would make more sense if it focussed purely on street homeless people who may not have that opportunity.<br>- Be more specific about your plans for dissemination as they are quite general. Which agencies, research groups and organisations will you communicate with?<br>- The study flow chart details only 100 participants with 50 in the intervention. Statistical comparisons would be underpowered in a sample of this size. Please justify your sample sizes against methodological and statistical assessment criteria and account for participant attrition in these plans.<br>- There needs to be detailed thought and resources put into participant attrition. Optimistically, four time period studies with homeless participants have a retention rate of around 40%.<br><br>Detailed budget summary:<br>The costs of this project seem astronomically high given the tiny number of participants that are being recruited into the study. Perhaps there is a more detailed budget that I cannot see but I would expect the budget to include staff costs, participant payments (which appear to be missing) and details of infrastructure expenditure and ad-hoc expenses. For staff costs each of the roles that will be needed should have a FTE salary, what proportion of FTE will be used, and a justification of that person’s time against the projects objectives.

NIHR | National Institute for Health Research

NIHR Health Services and Delivery Research Programme

RESEARCH PROPOSAL - EXTERNAL REVIEW OPEN COMMENTS

Comments
This project will be a real benefit for people that are experiencing homelessness ensuring they receive holistic care including health and social care. The project idea has to spread to the North West of England where there is a lot of homelessness in the cities that is not due to drug misuse, but just caught in the wheels of the benefit chaos. Reading this proposal shows that there is a connect between health and social care with a independent prescriber and social worker working together.<br><br>The project has a team that have worked together in the past on similar projects and have a proven record of success. The project is an extension of previous work but is working to build a larger RCT in the future.<br><br>This is the second read of this project. The authors have answered the questions and adjusted/revised the project as per the reviewers comments. This project is slightly shorter and fewer people which will be easier to manage and investigate outcomes of interest.

RESEARCH PROPOSAL - EXTERNAL REVIEW OPEN COMMENTS

Comments
This is an innovative and well written proposal. The project is well designed, with appropriate outcome measures, including an economic evaluation and process evaluation. The team is experienced in working in this area and with this intervention, and have worked together before.<br><br>--How will the research make a difference?--<br><br>The applicants make a strong case that people experiencing homelessness (PEH) have considerable unmet health and social care needs, and that their intervention is well placed to meet these needs. In particular, it builds on some of the widely recognised strengths of the community pharmacy sector, especially its accessibility both geographically and through care without appointments. In an age of remote appointments this seems even more valuable for PEH. The proposed intervention is holistic and based on the needs and priorities of individual PEH. Rather than seeing this as a "lack of clarity and focus" (a concern raised at stage 1), I see this as an appropriate patient-focused response to the heterogenous needs of this population. It is akin to approaches to social prescribing that are being ever more widely implemented. A successful PHOENIx intervention therefore has potential to improve the health and quality of life of PEH and to be widely implementable through the national community pharmacy network, so long as appropriate local voluntary sector partners were available. A pilot RCT is the next logical step in the rigorous development of PHOENIx and therefore therefore this funding application does have potential to lead to beneficial changes for PEH. There are also potential benefits to the health service, by reducing use of emergency services and making better use of the skills of community pharmacists (which has been a stated aim of governmental and professional policies for many years). In addition, my own (as yet unpublished) research indicates that greater opportunities for independent prescribing in community pharmacy are required to optimise its contribution to the NHS.<br><br>--Is the proposed research feasible from your perspective?--<br><br>The proposal seems to be largely feasible, although I do wonder whether 3 months is sufficient time to apply for and receive ethical approval and then set up study sites for such a potentially complex project. Feasibility will depend heavily on selecting appropriate community pharmacies which have contact with sufficient numbers of PEH. Based on the team's prior experience of working with PEH, it seems a safe assumption that they already have suitable sites in mind, although it would be reassuring to have their confirmation of this.<br><br>--What else could the applicants do to improve the research proposed?--<br><br>The team is very large (as raised at stage 1). I can see why this might be necessary with an intervention such as this, but I do wonder if some of the %FTE figures could be improved. 48% FTE (33% + 15%) for 22 months for statistical analysis seem a large figure, especially in comparison to the %FTE figures for other aspects of the application. Meanwhile only 4% FTE is dedicated to PPI.

Comments
The rationale for many of the stated outcomes is obvious, but it would strengthen the proposal if the applicants could provide further justification for why these particular patient reported outcomes were selected, especially those that relate to COPD.<br><br>Many of the proposed outcome measures are likely to be sensitive to adverse outcomes as well as positive outcomes, and I note that following concerns raised at stage 1 "Any adverse events will be collected by researchers and scrutinised by the OC/TMG". However, to ensure a balanced appraisal of the intervention, it would be helpful to see details of the structured collection and assessment of adverse events described in the detailed research plan. The process evaluation could also be specifically designed to seek information about unexpected adverse effects, which may not be health related.<br><br>I note that the independent prescribing pharmacists used in the study will be experienced (band 8a) and employed by the NHS. I do not doubt that this is a role that could be competently fulfilled by such staff. However, as many independent prescribing pharmacists specialise in just one clinical area, it would improve the proposal if the applicants could explain how pharmacists with suitable 'generalist' competence will be recruited and trained. The feasibility of doing this at scale will be an important consideration in the wider implementation of PHOENIx.

RESEARCH PROPOSAL - EXTERNAL REVIEW OPEN COMMENTS

Comments
Homelessness is clearly of great concern from a health and social care perspective and the challenges of effectively addressing health inequalities are widely acknowledged in the academic literature and wider Media, and are particularly pertinent since the COVID-19 pandemic. The potential impact of supporting some of the most vulnerable individuals in society is huge and the applicants clearly identify both the extent and seriousness of the homeless problem in the UK and propose an innovative means of effectively tackling what is a highly complex issue. The research team makes a compelling case for conducting the proposed work; extending the existing, yet under-utilized, NHS resource provided by community pharmacists. Evidence indicates that this group of health professionals are appropriately skilled, willing, and importantly, uniquely positioned to perform the role effectively in terms of helping this complex and marginalized patient group overcome barriers to healthcare. The applicants clearly indicate that overstretched traditional NHS services may not be suitable for this group and this intervention potentially offers an innovative method of addressing the issue.<br><br>Considerable preparatory work has been demonstrated by the applicants in support of conducting this feasibility work (and depending upon the results, proceeding to a large-scale trial of the intervention). The rationale for undertaking the work is clearly outlined and the novel PHOENIx intervention shows promising results to date. The fact that the intervention is provided in conjunction with an outreach worker and thus extends to include both work/social and health domains is impressive in terms of the wider societal context (helping with welfare benefits, volunteering roles etc) and ultimately offers an enhanced and more holistic service to this vulnerable group.<br><br>The research team has an excellent mix of skills. Both the project- and study oversight- management plans are robust. The applicants are to be commended on the quality and quantity of PPI, notably including the homeless charity heads as members of the study committees to provide vocal and honest input to inform the study.<br><br>The plan for recruitment is well described with a range of methods to advertise the study. One observation that may affect the study success is that opportunistic recruitment outlined will require the researchers to spend considerable time in each of the 4 pharmacies to maximise recruitment rates. Perhaps close communication with the pharmacist and outreach worker can facilitate recruitment, but personal experience of previously recruiting a vulnerable group of individuals in the community indicates that recruitment such as this proves to be extremely time-consuming, sporadic and requires the research team to be both flexible and ensure a visible profile to attract potential participants. Has the team considered the possibility of uneven recruitment across the four locations and how to proceed if one location has a high recruitment rate or if a location struggles to recruit?<br><br>The mixed methods study design proposed is appropriate to assess the feasibility. I am not an expert in qualitative research and thus am unable to comment on the proposed plan for analysis. However, it would be helpful to clarify the timing of the proposed interviews, i.e. will they be conducted during or after data collection? For example, will the intervention group participants be interviewed after the 6-month follow-up period? Would the applicants consider interviewing individuals from the control group? It would also be beneficial to outline which relevant health care professionals the researchers propose to interview- will this include the community pharmacists involved in providing the intervention and is it proposed to interview the outreach workers too? Their opinions would form an integral and valuable part of the feasibility evaluation and usefully inform the lessons to be learned.

Comments
Process evaluation and assessment of fidelity of the intervention appears to be sound. Economic evaluation described is also a valuable inclusion. The applicants acknowledge that this is a pragmatic study and recognize that the risk of contamination bias is largely unavoidable. A concern is that successful follow up of the control group may be particularly challenging, especially if the control is in contact with an individual receiving the intervention. Have the applicants considered how they might aim to successfully follow up at the time points other than offering the incentive? The financial incentive is appropriate, and experience has shown this is an effective way of reducing attrition. However, follow up at 3 and 6 months may be challenging, especially in maintaining contact with the control group. It would be helpful to clarify whether the outreach worker and pharmacist will play a role in assisting the researcher in terms of follow up and has a role to play in contacting or connecting with participants in either group.<br><br>Overall, this is an excellent study that is feasible and is extremely well justified. However, I have judged it good, simply on the basis of requiring minor points to be clarified or modified slightly.

RESEARCH PROPOSAL - EXTERNAL REVIEW OPEN COMMENTS

Comments
I structured my comments using the three guiding questions. In general, I think the proposal should be funded if minor changes are addressed or at least if the applicants thought about those points that are relevant when progressing to a potential definite trial.<br><br>How will the research make a difference?<br><br>The proposed research's goal is very worthy. Persons experiencing homelessness (PEH) are one of the most vulnerable groups in our society. Their situation is detrimental to their health and well-being and isolates them from the rest of us. In addition to these human costs, PEH can create an avoidable but significant cost burden to public services through expensive emergency care and mental health programs. Therefore, there is a clear ethical/moral argument and an economic one that supports interventions and prevention.<br><br>The proposed research will determine whether an integrated intervention delivered to PEH can be tested in a controlled way to measure the associated costs and benefits accurately. The integrated intervention - jointly delivered by prescribing pharmacists and a voluntary charity worker within participating community pharmacy - aims at dealing simultaneously with urgent health needs and social care needs such as housing or benefits. The applicants have delivered this integrated approach successfully for years, however, on a small scale. Given that the direct and indirect effects of COVID-19 increased the number of PEHs and the scale of the problem for existing PEHs, it is the right time to make the next step.<br><br>If the proposed research is successful, progression is made to the definite trial. In case (cost) effectiveness of the approach is demonstrated, this research may make a difference by benefiting a marginalised population and potentially reducing cone cost burden for the public purse.<br><br>Is the proposed research feasible from your perspective?<br>The proposed research is a pilot randomised controlled trial (RCT) that seems feasible to me. Using the intermediate step of a pilot trial backed by an experienced trial unit, the applicants chose the preferred path of careful progression. I want to comment on a few points, though:<br><br>The study flow chart is misleading as from the intervention, it jumps to the data collection of the three months follow up. I assume a baseline collection for all secondary outcomes, as mentioned in the full text.<br><br>The applicants plan for 60 minutes per PEH for the intervention. However, secondary data collection might increase this time (e.g. collection of EQ5D data might take up to 20 minutes per individual alone). Hence, more time (at least during the follow-ups) might be allocated per PEH.<br><br>When it should come to a definite trial, it may not be possible to analyse the integrated intervention's different factors. Therefore, the intervention may be a black box that does not disentangle the social determinants of health from the direct health effects of the prescribing part of the intervention. While this may only reflect the complex need of the targeted population, applicants may think about shining

Comments
more light on this issue in a potential definite trial.<br><br>It is unclear if the costs associated with the intervention will stay the same in the definite trial. For a potential definite trial, the cost components of the Intervention per PEH should be made explicit.<br><br>What else could the applicants do to improve the research proposed?<br><br>The applicants did, on average, a good job in addressing the questions from reviewers in stage 1. There are a few points that I find worth mentioning:<br><br>Firstly, the clarity about intervention purpose and focus (point 2 of stage 1 comments) could still be developed. I especially miss a clear long-term objective of the intervention. It is unclear if this is to build better therapeutic relationships between pharmacists and PEH, PEH progression to mainstream care, or getting the PEH out of homelessness eventually. This should be reflected in the endpoints of any definite trial.<br><br>The point above is linked to clarify the economic rationale. A starting point would estimate the cost burden to the NHS and broader public services due to PEH. The proposed intervention would better utilise pharmacists in the UK, who are under-utilised relatively to the associated training costs. Further, it might prevent costly care for PEH due to a lack of continuous care for this marginalised population. Finally, it might help lift PEH out of homelessness after all, which should be a positive return on investment per £ spent from the public purse.<br><br>Further, out of 17 applicants on the team, 10 (58%) contribute less than 5% of an FTE. I second the comments from stage 1 reviewers that the valuable contribution of those is questionable.<br><br>Finally, the health economic analysis within the definite trial should follow the "Methods for the development of NICE public health guidance" and is likely to rely on a cost consequence or cost-benefit analysis with a public sector perspective. QALYs will only be one outcome. The group should ensure that the chosen outcomes align with the objective function of the relevant decision-makers as an explicit decision criterion for evaluating the outcome of a CCA is lacking. Potentially the applicants may think about including blood pressure and BMI as two additional secondary outcomes as this would match basic measure on the Quality and Outcome Framework.

NIHR | National Institute for Health Research

NIHR Health Services and Delivery Research Programme

RESEARCH PROPOSAL - EXTERNAL REVIEW OPEN COMMENTS

**Comments**
As a person who has previously experienced homelessness, I found this proposal long overdue. It appears to be good value for money for it's potential benefits nationwide, given the inevitable suffering of marginalized persons already overlooked by most mainstream medicine. Obviously the often chaotic nature of Homeless People's lives preclude them from organizing appointments with GPs and accessing Healthcare within standard parameters, but does this automatically bar them from any intervention, I feel we should be embracing diversity and celebrate alternative lifestyles whether enforced by circumstances or by choice, this would obviously include all the people suffering mental health difficulties on the streets, through drugs and alcohol or through depression or psychosis. it's incredibly difficult for these patients to access care at the best of times but with the disadvantage of having a transitory base compounds an already insurmountable problem.<br><br>The element of risk of drop out has been addressed with the proposal to have sticky appointments and contributions from those in the charitable sector who marginalized people are often happier to see, and access services from, than official channels such as the medical and pharmacy services. On this note pharmacies are more accessible simply by there location and opening times, as well as ad hoc appointment provision would improve take up and continuation in this valuable study.<br><br>In all, what an incredibly splendid way to spend less than 800 thousand pounds, when this would make a huge impact on the fall out and suffering of the patients, families and friends, as well as the benefits of any form of research into alternative care pathways in general, which could be replicated for other client groups such as teenagers, psychiatric service users and older people.

RESEARCH PROPOSAL - EXTERNAL REVIEW OPEN COMMENTS

Comments

Applicant Response To External Review

Applicant Response

Reviewer 1

1.1 “I don’t understand the justification of such a large research team.”

People experiencing homelessness have complex health and social care problems, and their primary health and social care system is also complex. To transition smoothly to the definitive trial, the pilot study sites and team represent clinical and research expertise in the devolved environments. Lead clinicians will ensure adequate clinical input, governance, and to help shape policy. A large number of trialists and social/third sector staff from each site will ensure success.

1.2 “applicants need to better explain what they mean by recruiting a ‘wide team’.”

A large number of co-applicants.

1.3 “Unsure how ‘Paudyal collaborates closely with BCTU’ justifies 15% FTE.”

As the lead applicant Paudyal (alongside Lowrie) will be primarily responsible for all aspects of the study: governance, recruitment, data collection, analysis; and study sites, BCTU and NIHR liaison. The trial of this complexity requires extensive liaison with multiple sectors including community pharmacy, primary care, emergency departments, drug and alcohol services, social care services, charities, housing associations, prison and street intervention teams. Time is also needed to manage a large research team, undertake report writing, present interim and final study outcomes, and maintain external liaison. While the PHOENIx model of care was conceived and modelled in Glasgow, feasibility work involved Lowrie and Paudyal. Both have worked together on a number of projects.

1.4 “Relevant expertise – would be interested to see a track record of influencing policy and practice.”

In Glasgow, a RCT led by Mackie in primary care led to the setup of the UK’s first GP – Pharmacist service [1]. Lowrie and MacLaren then worked with Mackie to implement findings, going onto recruit, train and sustain 10, then 30 more pharmacists in GP practices in Glasgow. Qualitative work by Lowrie [2,3] helped consolidate, and the success of the Glasgow service led to rollout across the UK. Lowrie used the available evidence including relevant NICE, NSF and SIGN guidance, to secure funding for a specialist Glasgow Pharmacy Falls model of care which remains fully operational in Glasgow and influenced pharmacy practice in parts of England [4].

Lowrie led the Statin Outreach Support trial (PhD thesis) and this model of GP prescribing support was proven to work, leading to implementation of the model in Glasgow/Scotland [5].

The largest RCT to date in pharmacy was led by Lowrie; GP based pharmacist services specialising in Heart Failure in acute and primary care across the UK and North America [6].

PHOENIx service was conceived in Glasgow by Lowrie [7]. The service model has been adopted in

Applicant Response
Lothian, by one of the original PHOENIx team (Gibson).<br><br>Paudyal has led projects funded by Public Health England, local Health Boards and City Councils that have enabled understanding of disease epidemiology [8,9], barriers to accessing primary care [10], self care [11] medicines use [12] in persons experiencing homlessness, and understanding key players in transition of a person homelessness to a ‘housed’ life [13]. Through the joint funding from Public Health England and Sandwell Metropolitan Borough Council, Paudyal is currently evaluating presenting reasons and outcomes of Emergency Department and Inpatient hospital utilisation by all persons experiencing homelessness in England in the last 10 years by using Hospital Episode Statistics data. This work is expected to feed into national strategy around coding of homelessness when patients present to health services, and will allow to strengthen proactive prevention measures. This work is also complemented by an ongoing systematic review and meta-analysis [14]. Paudyal has previously successfully delivered (as the main study research fellow during his postdoctoral training) a comparative cost effectiveness evaluation of pharmacy based minor ailment services using prospective Cohort study comparing health and cost outcomes across pharmacy, general practices and Emergency Departments [15]. This work included study sites across England and Scotland. This work showed that £1 billion pa could be saved by expanding pharmacy minor ailment services. As a result, Minor Ailment Services is now a free service that can be accessed through vast majority of pharmacies across GB. This study also informed international trials [16,17].<br><br>1.5 “More explicitly stated ‘promising quantitative and qualitative outcomes’ from the feasibility<br><br>study may make this a more convincing proposal.”<br><br>A reduction in emergency department attendances and an increase in the number of scheduled outpatient appointment attendances in the PHOENIx intervention group. Independent qualitative research [18] concluded that informal, flexible and person-centred approach, and proactive immediacy of response (including provision of prescriptions), were critical ingredients in the initiation and maintenance of patient engagement, acting as a bridge to primary and secondary healthcare for people experiencing homelessness who are reluctant to utilise or physically unable to access alternative provision.<br><br>1.6 “The study presumes PEH will walk in and disclose a great deal of health and other personal information”<br><br>Most patients experiencing homelessness have multiple health problems and access community pharmacies for medicines supply and advice often on a daily basis [19,20]. There is already a high degree of trust between these patients and community pharmacy staff [Box 2 in the Stage 2 proposal]. While this currently may revolve around the provision of medicines (specifically, methadone/other opiate replacement therapy) in response to a prescription, we have found that patients tend to open up and disclose lots of health, social and other information in the context of sensitively conducted consultations with pharmacists, because of the role and their skills [18,20].<br><br>1.7 “It seems that PEH will be signposted, need to more explicitly state what the role is that pharmacists will play. Giving practical examples may be helpful.”<br><br>We rarely rely on signposting, because this does not tend to work for people experiencing homelessness. Instead we refer: phone to organise another service to accept the patient, and arrange

Applicant Response
handover, reminders and/or transport including taxis. Pharmacists lead on referral to different parts of the health service, and our collaborators lead on referrals to housing, social prescribing. Examples: addictions services for same day assessment and initiation of methadone; urgent referral to hospital (via ambulance). Our previous work [21] describes 13 different referral destinations in a sample of 52 patients.<br><br>1.8 "People very often have access to support and signposting through services and their inclusion thus seems to double up work."<br><br>Availability of, and access to health/social services is patchy in different accommodation venues and on the street. Even when available, patients have a choice as to who they engage with. Existing teams are stretched; our input in Glasgow has never been challenged on the basis of duplication of effort: patients' workers often cannot reach their patients, and ask that the PHOENIx team to track them down. We stick to patients, and know where they may be.<br><br>1.9 "Be more specific about your plans for dissemination"<br><br>We will share our findings with the Chief Pharmacists, Scottish Government Primary Care and homelessness leads, Community Pharmacy Scotland, Pharmaceutical Services Negotiation Committee in England and the leads of the main multiple chain pharmacies. Other venues for dissemination and engagement will be- Faculty of Homelessness and Inclusion Health, Shelter, Crisis, Homeless Link, Department of Housing, Community and Local Government, Department of Health, Royal Pharmaceutical Society, Royal College of GPs and Royal College of Emergency Medicine. Public Health England are participating in the research and will contextualise the research within current policy objectives [22], NHS Long Term Plan [23,24] and local government priorities. This research builds on these levers and the preventative focus of the Homelessness Reduction Act [22].<br><br>1.10 Statistical comparisons would be underpowered in a sample of this size."<br><br>Our aim is to assess if a definitive study is justified; we have no intention of using hypothesis testing to draw conclusions about the effectiveness of the intervention [25]. As recommended, we have justified our sample size on the basis of measuring uncertainty around our main feasibility measures of recruitment and retention [26,27].<br><br>1.11 "There needs to be detailed thought and resources put into participant attrition."<br><br>Our study has direct involvement of the street outreach teams; they are adept at reaching people through outreach, word of mouth, interactions with police and criminal justice workers, prison staff, visiting accommodation sites and walking the streets, links with case workers, accessing hospital records. Patients will also visit the pharmacy for collection of medicines, which offers another route to minimise attrition. We believe our multifaceted approach to follow up goes beyond attempts by previous teams.<br><br>1.12 "The costs of this project seem astronomically high"

Applicant Response
We acknowledge the costs are high, (described in detail in the application) and reasons why are in point 1 response above. We have made every effort to reduce the costs; the research team were cognisant of the complexity of the processes and rules around funding different parts of the study, and differences between Scotland/England. We welcome an opportunity to discuss any modifications to our funding if needed, should our study be approved.<br><br>Costs reflect the multi- sector and multidisciplinary and sticky nature of intervention required to recruit these patients. Our embedded health economics work uses collected resource use data together with outcome measures to indicate whether the intervention is likely to be cost-effective from a health service perspective, as well as exploring how a full-trial economic evaluation can best incorporate a wider societal (or at least public service perspective), beyond the NHS as others have done previously [28].<br><br>1.13 "The project idea has to spread to the North West of England"<br><br>We would be keen to explore inclusion study sites in North of England, for the reasons given.<br><br>Reviewer 2<br><br>2.1 "I do wonder whether 3 months is sufficient time to apply for and receive ethical approval and then set up study sites"<br><br>Lowrie, Paudyal and the research team will work together before receiving funding, if approved. Two Glasgow and one Birmingham pharmacy has already agreed to participate.<br><br>2.2 "48% FTE (33% + 15%) for 22 months for statistical analysis seem a large figure... only 4% FTE is dedicated to PPI."<br><br>We apologise for the error. The section 'justification of costs' reflects the true contributions of the statistics team costed in the proposal: Statistician (10% FTE for 22 months): expertise to allow interim and final analyses, short-term outcome papers and final reports; oversight by the senior statistician who is co- applicant at 5% FTE (22 months). Therefore the total is 10% + 5%=15%. We have checked the contributions of all other research team members and confirm that they are accurate.<br><br>2.3 "… justification for why these particular patient reported outcomes were selected, especially those that relate to COPD."<br><br>The main outcome of the pilot is to determine whether to proceed to a definitive trial. Secondary outcomes include a range of measures reflecting the complexity and number of patient's problems. We are assessing respiratory status, because of the importance, prevalence and mortality from COPD [8,29,30,31].

**Applicant Response**
2.4 “it would be helpful to see details of the structured collection of adverse events”<br><br>Assessment will mirror the health and social care check currently offered by PHOENIx team in Glasgow [8]. We will explore any adverse events to the process evaluation, as an open ended question.<br><br>2.5 “if the applicants could explain how pharmacists with suitable 'generalist' competence will be recruited and trained.”<br><br>Pharmacist prescribers initially obtain their prescribing qualification in one therapeutic area. Once in practice, they develop and broaden their skills. We already have a service specification for the PHOENIx as they have operated in Glasgow funded by short term service development grants. Requirements include the need to have experience or received training in multimorbidity. We will conduct a training needs assessment, and within the research / clinical team, organise additional training if necessary. The pharmacists will seek help from the clinicians involved in the study, just as they do in practice. We will recruit from the current pool of pharmacists in both cities, who already manage multimorbidity. From experience in Glasgow it is just as important to select pharmacists on the basis of their street sense, team ethos, non-judgemental approach, empathy.<br><br>2.6 “possibility of uneven recruitment across the four locations”<br><br>We have two other pharmacies in both sites ready to be enrolled should recruitment not proceed as planned.<br><br>2.7 “will interviews be conducted during or after data collection?”<br><br>We plan to interview patients at 3 months after their first contact in the intervention group. Patients in the control group will also be interviewed.<br><br>2.8 “Outline which relevant health care professionals the researchers propose to interview”<br><br>Stakeholders from addiction teams, GP practices, Simon Community Scotland, Sifa Fireside, mental health workers, caseworkers, Community pharmacy staff.<br><br>2.9 “how to aim to successfully follow up at the time points other than offering the incentive? …Clarify whether the outreach worker and pharmacist will play a role in assisting the researcher follow up.”<br><br>We have described ways in which we plan to follow up the control group, in response 1.11 above. Contamination is unavoidable due to 1:1 randomisation vs cluster randomisation. Process evaluation will assess whether contamination interferes with the intervention effect.<br><br>Reviewer 3

Applicant Response
3.1 "The study flow chart is misleading…it jumps to the data collection of the three months follow up."<br><br>Baseline assessment is mentioned in the flow chart: "100 consenting patients (50 Glasgow, 50 Birmingham) receive baseline assessment in pharmacy by researcher"<br><br>3.2 "more time (at least during the follow-ups) might be allocated per PEH."<br><br>This is assumption based on feasibility work. We can offer more time if needed, but 60 minutes is usually sufficient; patients with multiple health problems including problem substance use, often get restless if the consultation lasts more than 60 mins.<br><br>3.3 "the intervention may be a black box that does not disentangle the social determinants of health from the direct health effects of the prescribing…applicants may think about shining more light on this issue."<br><br>Strong synergistic interactions between different components, e.g. moving a patient off the street into accommodation, will impact on health. Qualitative process evaluation will explore these from the perspectives of the participants and stakeholders. Previous evaluation led by Paudyal [13] that specifically evaluated patient journey of transition from homelessness to a 'normal' life demonstrated the importance of dedicated homeless health services such as the proposed PHOENIx model that are easily accessible, tailored to their multiple complex needs.<br><br>3.4 "It is unclear if the costs will stay the same in the definite trial."<br><br>We will provide a detailed breakdown of the cost components, in line with recommended methodological practice for transparency and reproducibility of full economic evaluations [32,33].<br><br>3.5 "The team need to demonstrate how they are building on findings from the earlier feasibility study… I especially miss a clear long-term objective of the intervention."<br><br>The proposed RCT is based on comprehensive preparatory work: we delivered the intervention in different settings [7,21]; identified unmet need (under diagnosis/treatment) [8,29,30]; service access issues [7,10,12,21]; patients and stakeholders accepted our new care pathways [7,18,21]. Incorporated learning includes: careful choice of intervention staff for PHOENIx who have the street sense, team ethos, non-judgemental approach, empathy; immediate access to patient clinical records [34]. Having recruited, trained and trialled 7 pharmacists in the role during the feasibility work and services operating in Glasgow thus far, we learned that pharmacists can do this role well.<br><br>Patients supported progression to a trial. In particular, integrated established referral pathways and social prescribing. We have also incorporated patients and stakeholders' input to trial design, undertaken a systematic review [35] and updated in this Stage 2 application to ensure no duplication. The non-randomised feasibility study signalled improvements in outcomes and helped refine intervention duration and frequency (duration shortened to 6 months). The service development in a Glasgow city-centre pharmacy confirmed proof of concept. Patients had a median of 8-9 consultations over 5 months, however, the frequency was limited by the PHOENIx team due to high caseloads; patients were willing to be seen more frequently. Ideally, weekly contacts at the point of

Applicant Response
prescription collection.<br><br>The prevalence of problem drug use gave us an additional reason for a community pharmacy setting. In our recent national stakeholder event in Birmingham, interventions to prevent drug and alcohol related deaths emerged as one of three key priority areas [36]; also the need to offer interventions like the PHOENIx and an associated ask for integrated services with established referral pathways and social prescribing.<br><br>The long term objective is to improve patients' health.<br><br>3.6 "The point above is linked to clarify the economic rationale."<br><br>We fully expect for this work to quantify the cost burden borne by health and social care services (various outcome measures of relevance included), wider public services (e.g. prison encounters) and personal resource use (e.g. level of debt) specifically for participants in this study, in accordance with similar work [28]. However, we consider it would be duplication of effort to include an econometrics component, especially given that from a health and social care perspective at least, this burden has largely already been quantified within the existing literature using data linkage rather than prospective data collection [37,38].<br><br>3.7 "Further, out of 17 applicants on the team, 10 (58%) contribute less than 5% of an FTE."<br><br>We have offered a response to this point in our answer to Reviewer 1 (points 1.1 and 1.2).<br><br>3.8 "Potentially the applicants may think about including BP and BMI as two additional secondary outcomes"<br><br>Including economics in the feasibility trial prior to a definitive trial will allow to explore the most appropriate outcomes. Methods for the development of NICE public health guidance still allows for the use of QALYs and they are a relevant effectiveness measure (majority are health and social care outcomes) [39]. Existing economic evaluations in this area that have considered broader public sector perspectives have collected very similar outcomes [28]. We were relatively open to incorporating other measures that go beyond QALYs alluding to the potential need for a CCA. If a CCA is necessary we note the reviewer's point about the absence of explicit decision criterion for evaluating the outcome(s); it may be necessary to incorporate WTP methods in future, but we did not want to pre-empt that at this stage. Based on our collective experience of working in homeless health and care and in the third sector, BP and BMI do not seem to be relevant to the majority of participants. However these process outcomes will be captured as part of the researchers' assessments at follow up.<br><br>References<br><br>1.Mackie CA et al. Pharm J 1999;263: R7<br><br>2.MacRae F, Lowrie R et al. IJPP 2003;11:199-208.<br><br>3.Maclaren, Lowrie et al. IJPP 2003; 11: R7.

Applicant Response

4.Lowrie R. Pharmacy support to the falling elderly: from evidence base to model of care. Pharmacy Management. 2007.

5.Lowrie R et al. PLOS One 2014; doi:10.1371/journal.pone.0113370)

6.Lowrie R et al. Eur Heart J 2011; 33(3):314-324.)

7.Lowrie R et al. http://www.feantsa.org/download/march-2017-health-and-homelessness-newsletter2959399951318964653.pdf.

8.Bowen M, Paudyal V et al. BJGP 2018; 69 (685): e515-e525.

9.Paudyal V et al. Public Health 2021 (under review).

10.Gunner E, Paudyal V. BJGP 2019; 69 (685): e526-e536.

11.Paudyal V et al. Health Soc Care Comm 2020;28:160–172.

12.Paudyal V et al. Public Health 2017; 1;148:1-8.

13.Gibson-Smith K, Paudyal V et al. BJGP 2018; 68(667):e105-e113

14.Vohra N, Paudyal V et al. PROSPERO 2020; CRD42020189263.

15.Watson M, Paudyal V et al. BMJ Open 2015;5:e006261.

16.Dineen-Griffin et al. BMJ Qual Saf 2020; 1;29(11):921-31

17.Rafferty et al. Cost Eff Resour Alloc 2017; 15:3.

18.Johnsen S et al. BMC Health Serv Res 2021;21(1):1.

19.Paudyal V et al. Int J Clin Pharm 2019;15;41:1:215-27.

20.Jagpal P, Paudyal V et al. Pharmacy 2019, 7, 153.

21.Lowrie F, Lowrie R et al. Int J Pharm Pract 2019. 27(4):355-61.

22.Paudyal V. The Lancet 2018;21;392(10143):195-7.

23.www.longtermplan.nhs.uk

24.https://www.england.nhs.uk/blog/future-plans-for-the-pharmacy-workforce/.

25.Lancaster GA et al. J Eval Clin Pract 2004;10(2):307-12.

26.Lancaster GA. Pilot Feasibility Stud 2015;1:1.

27.Hooper R. NIHR RDS communication.

28.Cornes et al (NIHR). https://www.journalslibrary.nihr.ac.uk/programmes/hsdr/1315610/#/.

29.Queen A, Lowrie R et al. BJGP 2016 doi:10.3399/bjgpopen17X100941.

30.Zeitler M, Lowrie R et al. J Prim Care Comm Health 2020;11:1-13.

31.Aldridge et al. https://wellcomeopenresearch.org/articles/4-49.

Applicant Response
32.Caro JJ et al. Med Decis Making 2012;32;5:667-77.<br><br>33.Husereau D et al. Value Health 2013;16;2:231-50.<br><br>34.Lowrie R, Paudyal V et al. Int J Eq Health 2021; 20;1:1-3.<br><br>35.Hanlon P, Lowrie R et al. BMJ Open 2018;8(4):e020161.<br><br>36.Jagpal P, Paudyal V et al. Int J Equity Health 2020. Doi:10.1186/s12939-020-01206-3<br><br>37.https://www.gov.scot/publications/health-homelessness-scotland/<br><br>38.McCormick B et al. Clin Med 2016;16(6):506.<br><br>39.https://www.nice.org.uk/process/pmg4/chapter/incorporating-health-economics
