# Supplementary material for: Protocol for a pilot randomised controlled trial to evaluate integrated support from pharmacist independent prescriber and third sector worker for people experiencing homelessness: the PHOENIx community pharmacy study
Source: Pilot Feasibility Stud. 2023 Feb 23;9:29. doi: 10.1186/s40814-023-01261-x (PMC9946705; doi:10.1186/s40814-023-01261-x)
Supplement: Supplementary file 4 — Additional file 4. Peer review reports. [file 40814_2023_1261_MOESM4_ESM.docx]

**Please list the feedback received at first stage and under separate headings indicate what has changed as a result. Please describe and explain any additional changes that have been made to this proposal since the stage 1 application e.g. in the light of new research.**

1. Demonstration of how we built on findings from feasibility work.

Comprehensive preparatory work was necessary. Different pharmacists/third sector workers delivered the intervention in different settings [1-3]. We identified unmet need (under diagnosis/treatment) [4-6]; service access issues [1,2,7,8]; patients and stakeholders accepted our new care pathways [1,2,9]. Learnings incorporated includes:

-careful choice of intervention staff for PHOENIx

- immediate access to patient clinical records and expert advice needed

-patients supported the intervention for progression to a trial. In particular, integrated established referral pathways and social prescribing.

-patients and stakeholders' input to trial design

-systematic review and update in this Stage 2 application to ensure no duplication [10]

-non randomised feasibility study signalled improvement [11] and helped refine intervention duration and frequency (duration shortened to 6 months)

-service development in a Glasgow city-centre pharmacy confirmed proof of concept

2. Lack of clarity about intervention purpose and focus

Our aim is to engage with people who are homeless, then assess and address housing, health, benefits and social activities. While there are common areas of intervention e.g. addictions, respiratory, the intervention is not focussed on any particular problem a-priori because our target group have multiple health conditions in addition to a pressing need for housing, benefits, and social activities. Only the patient can say which one is most important: patients prioritise and we help them to tackle one problem at a time. Patients and stakeholders endorse this. We have revised our intervention description for Stage 2.

3. Concerns about focus of prescribing medicines in isolation from wider health problems.

Prescribing is one of many components of our intervention. If we do prescribe after a full health assessment, complex needs are itemised, and the patient, pharmacist and third sector worker agree priorities. Established, immediate referral pathways will be followed for conditions requiring specialist input, e.g. mental health crisis.

4. Greater attention to potential harms and safety issues.

To date, we have not encountered any harms. Clinical decisions including prescribing and referral are made by the band 8a pharmacist. GP or other expert advice is available (shared electronic clinical records are accessed before clinical interventions). No treatments are investigational. Clinical governance is provided by the specialist Homeless health service GP teams. Any adverse events will be collected by researchers and scrutinised by the OC/TMG.

5. Concerns about cost/ duration of intervention, and the sustainability of the intervention and associated NHS Support and treatment costs.

We have revised cost allocations and given the complexity of the intervention and multi-sector involvement, our proposed costs we hope represents excellent value for money. For stage 2 we have maximised chances of sustainability by reducing the duration of the intervention from 10 to 6 months, and duration of trial from 26 to 22 months.

6. Possible bias through signposting the control group to ED/ visits as a primary outcome.

We plan a pragmatic trial where this type of bias is unavoidable. If researchers encounter a patient who needs urgent medical care referral to a GP or ED may be needed. The primary outcome remains whether we should proceed to a definitive trial.

7. Health element more focussed on the population being studied through the inclusion of outcomes relevant to substance misuse

We have included more outcome markers for improved access to, and uptake of services and treatments for people with problem drug use in the stage 2 application

8. Practical questions (population transience/seasonality)

From our experience of delivering the intervention, we find long term homelessness is common amongst service users. Our partners (Simon Community Scotland and Sifa Fireside) are street based third sector organisations, adept at case finding, and building relationships, and can use their networks to enhance follow up, retention and maximise engagement. Tracking patients from community into prison will be challenging, we will explore how to retain data collection intervals as part of the pilot study. Seasonality of need is not something we have encountered in homelessness to date.

9. Ethical issues: potential dependence on time limited services.

In our experience of pragmatic intervention trials, dependence among recruited patients has not surfaced previously. Patients will be reminded of the fixed duration of intervention and signposted to their GP/community pharmacy as usual. If results are favourable as per our set criteria, we will move swiftly to a definitive trial, which may show clinical/cost effectiveness, arguing for continuing availability of PHOENIx.

10. Progression criteria look ambitious (80% data completeness), given anticipated problems in collection and attrition

We have reduced to 50-70%.

11. Low FTE contribution of multiple co-applicants; the team are asked to demonstrate the meaningful contribution of each.

We have asked two people to go onto the bench, and increased the commitment of others. Team reflects integrated nature of the proposed intervention.

12. Consideration of how this proposal links to and complements other similar studies in the HS&DR portfolio would be helpful.

The NIHR resource suggested here [12] will be included in pharmacist training. Study by Cornes et al. [13] tested a realist hypothesis, reinforcing practical support e.g. health and housing; including an economic analyses where one model took a wider societal perspective. We have experience collecting EQ5D5L data. Crane (2016) [14] targeted young adults, who have different care pathways in Glasgow and Birmingham.

References to this section are separately listed in the attachment- ‘References’.

1. Lowrie F,**Lowrie R** et al. A descriptive study of a novel pharmacist led health outreach service for those experiencing homelessness. Int J Pharm Pract. 2019. 27(4):355-61.
2. **Lowrie R**, **Williamson A**, Spencer R, Hair A, Gallacher I, Hewitt N. Collaborative engagement for long term conditions by clinical pharmacists for people who are homeless in Glasgow, Scotland http://www.feantsa.org/download/march-2017-health-and-homelessness-newsletter2959399951318964653.pdf.Accessed 02 Feb 2021.
3. **Lowrie R**, Hair A et al. How pharmacists are helping the homeless in Glasgow. Pharm J 2017 (May). DOI: 10.1211/CP.2017.20202677
4. Queen AB, **Lowrie R** et al. Multimorbidity, disadvantage, and patient engagement within a specialist homeless health service in the UK: an in-depth study of general practice data. Br J Gen Pract Open. 2017;1:3:bjgpopen17X100941.
5. Bowen M, **Paudyal V** et al. Multimorbidity and emergency department visits by a homeless population: a database study inspecialist general practice. Brit J Gen Pract. 69 (685): e515-e525.
6. Zeitler, M., Williamson, A.E., Budd, J., Spencer, R., Queen, A. and Lowrie, R., 2020. Comparing the impact of primary care practice design in two inner city UK homelessness services. Journal of Primary Care & Community Health, https://doi.org/10.1177/2150132720910568.
7. Gunner E, **Paudyal V** et al. Perspectives of homeless individuals on the provision and accessibility of primary healthcare services: a qualitative study. Br J Gen Pract. 2019; 69 (685): e526-e536.
8. **Paudyal V**, MacLure K et al.. ‘When you are homeless, you are not thinking about your medication, but your food, shelter or heat for the night’: behavioural determinants of homeless patients' adherence to prescribed medicines. Public Health. 2017; 1;148:1-8.
9. Johnsen S, Cuthill F, Blenkinsopp J. Qualitative Evaluation of Clinical Pharmacist Prescribing Input into the Care of People Experiencing Homelessness. Institute for Social Policy, Housing and Equalities Research: Edinburgh, UK. 2019.
10. Lowrie R, Stock K, Lucey S, Knapp M, Williamson A, Montgomery M, Lombard C, Maguire D, Allan R, Blair R, Paudyal V. Pharmacist led homeless outreach engagement and non-medical independent prescribing (Rx)(PHOENIx) intervention for people experiencing homelessness: a non-randomised feasibility study. International Journal for Equity in Health. 2021 ; 20(1):1-3. <https://doi.org/10.1186/s12939-020-01337-7>
11. Hanlon P, **Lowrie R** et al. A systematic review of interventions by healthcare professionals to improve management of noncommunicable diseases and communicable diseases requiring long-term care in adults who are homeless. BMJ Open 2018;8(4):e020161.
12. National Institute for Health Research (2020) Improving inclusion of under-served groups in clinical research: Guidance from the NIHR INCLUDE project. UK: National Institute for Health Research. Available at: [www.nihr.ac.uk/documents/improving-inclusion-of-under-served-groups-in-clinical-research-guidance-from-include-project/25435](https://www.nihr.ac.uk/documents/improving-inclusion-of-under-served-groups-in-clinical-research-guidance-from-include-project/25435) (Accessed 04 Feb 2021)
13. National Institute for Health Research (2015). Effectiveness and Cost-effectiveness of 'Usual Care' Versus 'Specialist Integrated Care': A Comparative Study of Hospital Discharge Arrangements for Homeless People in England. Available at:  <https://www.journalslibrary.nihr.ac.uk/programmes/hsdr/1315610/#/> (Accessed 04 Feb 2021)
14. Crane M, Joly L, Manthorpe, J. (2016) Rebuilding Lives: Formerly homeless people’s experiences of independent living and their longer term outcomes. London: The Policy Institute at King’s/Social Care Workforce Research Unit.
